# Enhanced Thermoelectric Performance of Tin(II) Sulfide Thin Films Prepared by Aerosol Assisted Chemical Vapor Deposition

**DOI:** 10.1021/acsaem.3c00608

**Published:** 2023-04-03

**Authors:** Yu Liu, Paul D. McNaughter, Feridoon Azough, Xiaodong Liu, Jonathan M. Skelton, Andrey V. Kretinin, David J. Lewis, Robert Freer

**Affiliations:** † Department of Materials, University of Manchester, Oxford Road, Manchester, M13 9PL, U.K.; ‡ Department of Chemistry, University of Manchester, Oxford Road, Manchester, M13 9PL, U.K.; § National Graphene Institute, University of Manchester, Oxford Road, Manchester, M13 9PL, U.K.

**Keywords:** SnS, thin film, thermoelectric, aerosol-assisted chemical vapor deposition (AACVD), density-functional theory (DFT)

## Abstract

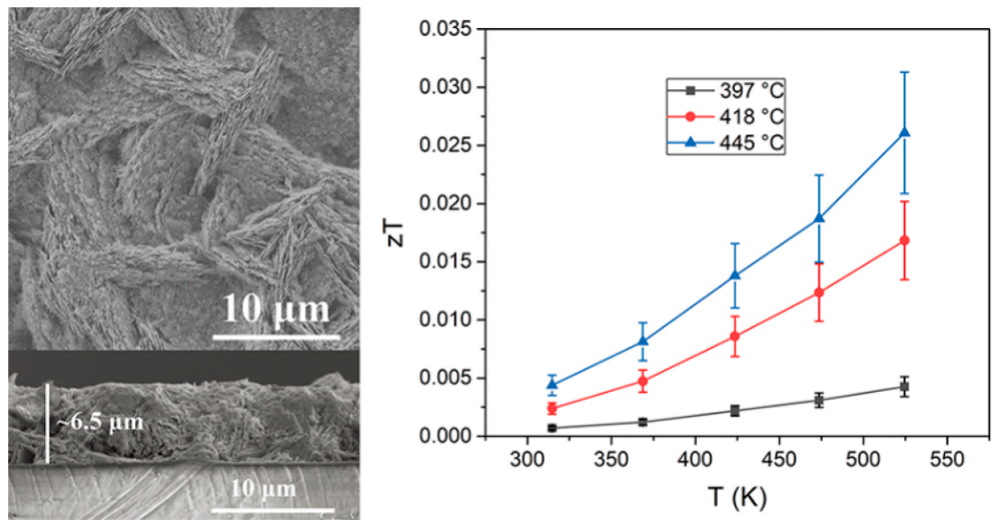

Orthorhombic SnS exhibits excellent thermoelectric performance as a consequence its relatively high Seebeck coefficient and low thermal conductivity. In the present work, polycrystalline orthorhombic SnS thin films were prepared by aerosol-assisted chemical vapor deposition (AACVD) using the single source precursor dibutyl-*bis*(diethyldithiocarbamato)tin(IV) [Sn(C_4_H_9_)_2_(S_2_CN(C_2_H_5_)_2_)_2_]. We examined the effects of the processing parameters on the composition, microstructure, and electrical transport properties of the SnS films. Deposition temperature dominates charge transport; the room temperature electrical conductivity increased from 0.003 to 0.19 S·cm^–1^ as deposition temperature increased from 375 to 445 °C. Similarly, the maximum power factor (PF) increased with deposition temperature, reaching ∼0.22 μW·cm^–1^·K^–2^ at 570 K. The power factors for SnS films deposited by AACVD are higher than values from earlier work on SnS bulks and SnS/SnSe films at temperatures up to 520 K. The electronic structure and electrical transport properties of SnS were investigated using density-functional theory to provide an improved understanding of the materials performance. To the best of our knowledge, the thermal conductivity (κ) of SnS film was measured for the first time allowing the figure of merit (*zT*) for SnS film to be evaluated. A relatively low thermal conductivity of ∼0.41 W·m^–1^·K^–1^ was obtained at 550 K for SnS films deposited at 445 °C; the corresponding *zT* value was ∼0.026. The SnS films are good candidates for thermoelectric applications and AACVD is a promising technique for the preparation of high-performance thermoelectric films.

## 1 Introduction

As we seek to address the growing need for renewable energy sources, thermoelectric (TE) materials have attracted considerable interest. TE materials enable the conversion between thermal energy and electrical energy and the opportunity to generate power from waste heat.^1^ The thermoelectric performance of a candidate material is described by the thermoelectric figure of merit (*zT*) which is defined in eq 1
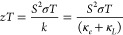
1where *S* is the Seebeck coefficient, σ is the electrical conductivity, κ is the thermal conductivity (which is the sum of electronic thermal conductivity (*κ*_*e*_) and lattice thermal conductivity (*κ*_*L*_)), *T* is the absolute temperature. The product *S*^2^ σ is defined as the power factor (PF).^2^ In order to maximize *zT*, the focus is to increase PF by increasing the Seebeck coefficient and/or electrical conductivity, and to reduce thermal conductivity. This is problematic in bulk materials due to the entanglement of electronic and thermal transport parameters embodied by the Wiedmann–Franz law. However, strategies to circumvent this limitation have been developed and include grain boundary engineering,^3^ and compositional control (e.g., doping, engineered inclusions, and alloying).^4^ All of these strategies rely on optimizing thermoelectric performance by tuning the carrier concentration and reaching the optimum balance between Seebeck coefficient, electrical conductivity and electronic thermal conductivity to maximize *zT*. Band structure engineering, such as band convergence and resonant level incorporation near the band edge has been also used to improve Seebeck coefficients without degrading electrical conductivity.^5^ Lattice thermal conductivity is relatively independent of the electrical conductivity, so reducing the lattice thermal conductivity is also an effective way to optimize *zT*.^6^ This can be achieved by enhancing phonon scattering through nanostructuring, and control of point defects and dislocations.^7^

Tin sulfide (SnS) is a IV–VI semiconducting compound, which crystallizes in an orthorhombic structure (*Pnma*/*Pbnm* phase) at room temperature, transforming into *Cmcm*/*Bbmm* phase around 878 K.^8^ SnS is an attractive TE material because it is an inexpensive, nontoxic, and earth-abundant semiconductor.^9^ Furthermore, it possesses a high Seebeck coefficient and low lattice thermal conductivity due to its layered structure.^10^ The electrical conductivity of pure SnS is too low for device applications, but it can be enhanced by adjusting carrier concentration.^11,12^ The thermoelectric properties of single crystal and ceramic SnS have been the subject of a number of investigations. Wu et al.^13^ grew SnS and Na-doped SnS single crystals and showed thermoelectric performance was enhanced by Na doping, increasing the maximum *zT* from ∼0.35 for pure SnS to 1.1 for Na_0.02_Sn_0.98_S at 870 K. Later, He et al.^14^ reported that thermoelectric properties of SnS crystal could also be enhanced by Se alloying; the maximum *zT* at 873 K was increased to ∼1.6 for SnS_0.91_Se_0.09_. Polycrystalline SnS bulk ceramics were prepared by Tan et al.^15^ using mechanical alloying and spark plasma sintering (SPS); the highest *zT* was 0.16 at 823 K. SnS bulk ceramics, containing rod-shaped nanocrystals, were synthesized by rapid annealing, achieving a maximum *zT* of 0.25 at 773 K.^16^ Dopants have also been introduced into polycrystalline SnS bulk ceramics to improve electrical transport properties and reduce thermal conductivity. Zhou et al.^12^ doped polycrystalline SnS with Na to increase hole concentration, achieving a peak *zT* value of 0.65 at 850 K. Han et al.^17^ prepared SnS_1–*x*_Se_*x*_ (0 < *x* < 1) solid solutions and obtained a maximum *zT* of 0.82 for SnS_0.2_Se_0.8_ at 823 K; defects in the solid solutions acted as phonon scattering centers, reducing the lattice thermal conductivity. By using Ag doping (0.5%), Tan et al.^11^ reported an improved *zT* value of 0.6 at 873 K. Later, a maximum *zT* value of 1.1 at 877 K was achieved by Ag doping and vacancy engineering for polycrystalline Sn_0.99_Ag_0.05_S by Asfandiyar et al.^18^ A similar high *zT* of ∼1.1 was reported by Wu et al.^19^ for Na_0.02_Sn_0.98_S_0.5_Se_0.5_ at 820 K.

To address the requirement for low power and portable devices for microgeneration, there is growing interest in thin film thermoelectrics.^20^ The small scale, lightweight films offer routes to enhance the thermoelectric performance through a range of preparation routes. Moreover, the interface between the film and the substrate, or the ones between multilayers, can exert significant effects on grain growth and film properties.^21^ However, compared to extensive work on bulk SnS thermoelectric materials, there have been relatively few investigations of SnS thin films. Robinson et al.^22^ prepared SnS thin films by low pressure chemical vapor deposition (LPCVD), achieving a maximum PF value of 0.049 μW cm^–1^·K^–2^ at 450 K. However, the thermal conductivity and *zT* of the films were not measured, and to the best of our knowledge, to date there is no documented experimental research on these parameters. In recent work, SnS thin films were deposited for photovoltaic, supercapacitor, and solar cell applications by a variety of techniques including thermal evaporation,^23^ radio frequency magnetron sputtering,^24^ modified chemical vapor deposition (CVD),^25^ spray pyrolysis, spin coating,^26^ chemical bath deposition (CBD),^27^ and electrodeposition.^28^ Among the available deposition methods, aerosol-assisted chemical vapor deposition (AACVD), a variant of CVD, is a low-cost and simple technique which can be conducted under ambient pressure for the preparation of uniform films with the extra advantage of high purity and reproducibility.^29^ AACVD also reduces the volatility requirement for precursors and allows the deposition of films from single-source precursors (SSPs), making it easier to control stoichiometry and morphology by designing the precursors.^30^ Kevin et al.^29^ synthesized SnS thin films containing sheet-like grains by AACVD using tin(II) precursors with the formula [Sn(S_2_CNRR′)_2_] (where R = Et, R′ = *n*-Bu (**1**); R = Me, R′ = *n*-Bu (**2**); R = R′ = Et (**3**)). Ramasamy et al.^25^ successfully deposited SnS thin films using a range of organotin dithiocarbamates by AACVD.

In this work, we prepared SnS films by AACVD using dibutylbis(diethyldithiocarbamato)tin(IV), [Sn((C_4_H_9_)_2_S_2_CN(C_2_H_5_)_2_)_2_], as single-source precursor and optimized the AACVD deposition parameters (flow rate, solution concentration, and deposition temperature) to enhance charge transport properties. For the first time, the effects of AACVD deposition parameters on the growth of SnS films are systematically investigated. We conclusively show that the deposition temperature has the most influence on morphology and electrical conductivity. For films measured below 540 K, we achieved the highest PF values reported for SnS thin films. The thermal conductivity of SnS films was measured for the first time, and the *zT* values were evaluated. The electronic structure and thermoelectric performance of bulk SnS were also investigated using density functional theory calculations to provide an improved understanding of the materials and processes.

## 2 Materials and Methods

### 2.1 Experimental Methods

#### 2.1.1 Synthesis and Characterization of Precursors

##### General Considerations

All reagents and solvents (detailed in the synthesis and deposition sections below) were obtained from Sigma-Aldrich and used without further purification. The preparation of dibutyl-*bis*(diethyldithiocarbamato) tin(IV) [Sn(C_4_H_9_)_2_(S_2_CN(C_2_H_5_)_2_)_2_] was conducted under an inert atmosphere of dry nitrogen using a Schlenk line.

##### Characterization of Precursors

Elemental analysis, to confirm the purity of the precursors, was carried out by the microanalytical laboratory of The University of Manchester. Analysis of C, H, N, and S analysis was carried out using a Thermo Flash 2000 and the Sn content determined by inductively coupled plasma atomic emission spectroscopy (ICP-AES) analysis using a Thermo Scientific iCAP 6300 DUO. Thermogravimetric analysis (TGA) and differential scanning calorimetry (DSC), were carried out by a TGA/DSC analysis Mettler-Toledo from 10 to 600 °C with a heating rate of 10 °C·min^–1^ under nitrogen.

##### Synthesis of Dibutyl-*bis*(diethyldithiocarbamato) tin(IV) [Sn(C_4_H_9_)_2_(S_2_CN(C_2_H_5_)_2_)_2_]

First, 0.03 mol (6.8 g) of sodium diethyldithiocarbamate trihydrate (ACS reagent, purity ≥99.0%) and 0.015 mol (4.6 g) dibutyl dichloride tin(IV) (purity 96%) were dissolved separately in ethanol, and the solutions were mixed and stirred for 1 h. The fine, white sodium chloride powder generated during stirring was removed by vacuum filtration and the pale-yellow filtrate distilled under vacuum until around 50 mL of solution was left. The solution was kept at 4 °C for 12 h for recrystallization; colorless crystals were formed. The crystals were isolated by vacuum filtration, washed three times in 30 mL of cold ethanol and dried at room temperature under vacuum. Yield: 6.1 g (77%). Anal. Calcd for C_18_H_38_N_2_S_4_Sn: C, 40.83; H, 7.23; N, 5.29; S, 24.22; Sn, 22.42. Found: C, 40.86; H, 7.30; N, 5.28; S, 24.08; Sn, 22.43.

#### 2.1.2 Deposition of SnS Films by AACVD

Prior to deposition, glass substrates (1.5 cm × 2.5 cm) were cleaned and sonicated in acetone for 30 min and were then loaded into a reactor glass tube after drying by compressed air. The precursor [Sn(C_4_H_9_)_2_(S_2_CN(C_2_H_5_)_2_)_2_] was dissolved in 10 mL of toluene in a two-neck flask and stirred for 30 min before use. The reactor glass tube was placed in a preheated Carbolite tube furnace connected to the flask. Aerosol droplets were generated by nebulizing the precursor solution using a digital ultrasonic humidifier. The precursor-infused aerosol was transferred into the reactor tube using argon (Ar) gas, delivered with a constant flow rate (150 to 300 cm^3^·min^–1^). By evaporation of the solvent and thermal decomposition of the precursor, films were deposited onto the substrates. The temperature profile for the Carbolite tube furnace (Figure S1) was measured using a K-type thermocouple under Ar gas flowing at a rate of 150 cm^3^·min^–1^. With the ability to control the gas flow rate, precursor solution concentration, and deposition temperature, it was possible to independently examine their effects on the composition, microstructure, and thermoelectric performance of the SnS films.

#### 2.1.3 Characterization of the SnS Films

Grazing incidence X-ray diffraction (GIXRD) was performed on SnS films using a PANaytical X’Pert Pro Diffractometer with a Cu Kα source (λ = 1.540598 Å) at an incidence angle of 3°. Phase identification was carried out by X’Pert Highscore Plus; lattice parameters were determined by Rietveld refinement using the TOPAS computer program.^31,32^ The quality of refinement was verified by the *R* weighted profile (*R*_wp_) and goodness of fit (GOF). The microstructures and chemical compositions of SnS films were examined using a Tescan MIRA3 FEG-SEM equipped with an energy-dispersive X-ray (EDX) detector. Film thicknesses were determined from cross-sectional SEM images. The areal surface porosities for the thin films were estimated by ImageJ. Transmission electron microscopy (TEM) images and selected area electron diffraction (SAED) patterns were acquired by a FEI Tecnai 20 TEM equipped with a LaB6 electron gun operated at 200 kV. High-resolution transmission electron microscopy (HRTEM) images were collected on a FEI Tecnai F30 FEG TEM operated at 300 kV. TEM samples were prepared by removing flakes of the film from the glass substrate, grinding in a mortar and pestle and dispersing in 2-propanol. After sonication, the suspension was dropped onto a commercial holey, carbon-coated copper grid and dried.

Sheet resistance was measured using an Ossila 4-point probe station; resistivity was determined from the sheet resistance and film thickness; uncertainties in data are taken to be 1%. The optical bandgap of the SnS films was determined from UV–vis–NIR absorbance spectra collected on a PerkinElmer Lambda 1050 UV/vis/NIR spectrometer. Raman spectra were obtained using a Horiba LabRam HR Evolution system with a 633 nm laser. In-plane electrical conductivity, Seebeck coefficients, and power factor were determined by an ULVAC ZEM-3 (330–570 K) under low-pressure helium (He) atmosphere. The data uncertainties for electrical conductivity, Seebeck coefficients, and PF were 3%, 5%, and 10% respectively. The in-plane thermal conductivity of the SnS film deposited at 445 °C was determined from 300–550 K by the 3 Omega technique under vacuum using a Linseis Thin Film Analyzer (TFA).^33,34^ As conductive samples are required for thermal conductivity measurement by TFA and as SnS films deposited on the test chip at low temperatures exhibited low conductivity, the SnS films prepared at 445 °C, with the highest electrical conductivity, were chosen for the thermal conductivity measurement. For the measurements, the SnS film was deposited by AACVD on a test chip containing a silicon nitride (Si_3_N_4_) membrane (Figure S2);^33^ a higher than normal precursor solution of 20 mL and a higher solution concentration of 0.06 mol·L^–1^ were used to overcome the difficulties encountered in depositing the SnS film on the chip. The in-plane *zT* values for the SnS films deposited at 397, 418, and 445 °C were determined from the measured thermal conductivities and power factor values. The uncertainty in the thermal conductivity data is 10%. The resulting uncertainty in *zT* is approximately 20%.

### 2.2 Computational Modeling

The electronic structure of bulk SnS in the orthorhombic *Pbnm* structure was investigated using first-principles calculations with density-functional theory (DFT). The calculations employed the pseudopotential plane-wave DFT formalism implemented in the Quantum ESPRESSO (QE) package.^35,36^ The Perdew–Burke–Ernzerhof (PBE) generalized gradient approximation (GGA) functional was used to describe the electron exchange and correlation.^37^ Ultrasoft pseudopotentials were employed to model the nuclei and core electrons,^38,39^ with 14 valence electrons for Sn (5s^2^ 5p^2^ 4d^10^) and six for S (3s^2^ 3p^4^). Based on explicit convergence tests (Figure S3) a kinetic energy cutoff of 75 Ry for the plane-wave basis (1 Ry = 13.6 eV) and a uniform *k*-point sampling mesh with 8 × 3 × 9 subdivisions were selected for the calculations. For ultrasoft pseudopotentials, the cutoff for representing the electronic charge density (ecutrho in QE) needs to be ∼10× larger than the cutoff for the wave functions (ecutwfc), and thus a cutoff of 750 Ry was employed for the charge density. The structure of SnS was optimized to tolerances of 10^–6^ Ry and 10^–5^ Ry bohr^–1^ on the total energy and forces, respectively, and a tolerance of 10^–6^ Ry was applied to the total energy during the electronic self-consistent-field (SCF) cycle.

To predict the electrical transport properties, the Seebeck coefficients, electrical conductivity and power factor of bulk SnS in the range of 300–850 K were estimated using semiclassical Boltzmann transport theory within the constant-relaxation-time approximation, as implemented in the BoltzTraP package.^40^ An additional *k*-point convergence test was conducted for these calculations since a finer sampling mesh is generally required. As shown in Figure S4, we found that the calculated Seebeck coefficients converged with a 10 × 4 × 11 mesh. The *lpfac* parameter (number of lattice points per *k*-point) was set to 5 in the BoltzTraP calculations as we found negligible difference between these Seebeck coefficients obtained with different values. A constant relaxation time (τ) of 1.51 × 10^–15^ s taken from the literature,^41^ derived from experimental data on bulk SnS, was used to determine the electrical conductivity.

## 3 Results and Discussion

### 3.1 Electrical Properties of *Pbnm* SnS from Density Functional Theory

In order to provide a reference point for our thin-film measurements, we performed a set of first-principles DFT calculations to investigate the electronic structure and transport properties of bulk SnS.

**Figure 1:**
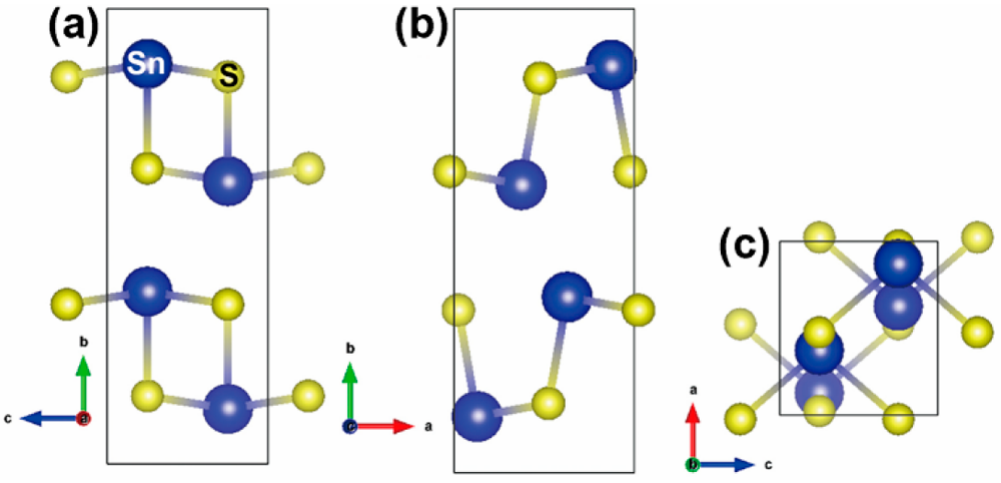
Optimized crystal structure of SnS from first-principles DFT calculations. The structure is shown along the (a) *a*, (b) *b*, and (c) *c* axes of the unit cell. The unit cell is orthorhombic (*Pbnm*) with lattice parameters *a* = 4.429 Å, *b* = 11.419 Å, and *c* = 4.028 Å and α = β = γ = 90°. These images were generated using VESTA.^46^

Figure 1 shows the optimized structure of SnS. In the *Pbnm* spacegroup setting, the *b* axis is chosen as the longest axis, which is distinct from the *Pnma* group setting used in some literature in which the *a* axis is chosen as the longest axis. Both settings correspond to the same crystal symmetry.^42^ In the *Pbnm* structure, each Sn atom is bonded to three S atoms to create a pseudo-2D network that extends along the *a* and *c* directions. Each Sn atom has a fourth coordination site occupied by stereochemically active Sn 5s lone pairs of electrons, which projects into the interlayer space and results in weak van der Waals coupling along the *b* direction.^43,44^ The optimized lattice constants are *a* = 4.429 Å, *b* = 11.419 Å, and *c* = 4.028 Å, which are consistent with similar calculations,^45^ and experimental results on bulk SnS.^11,17^

**Figure 2:**
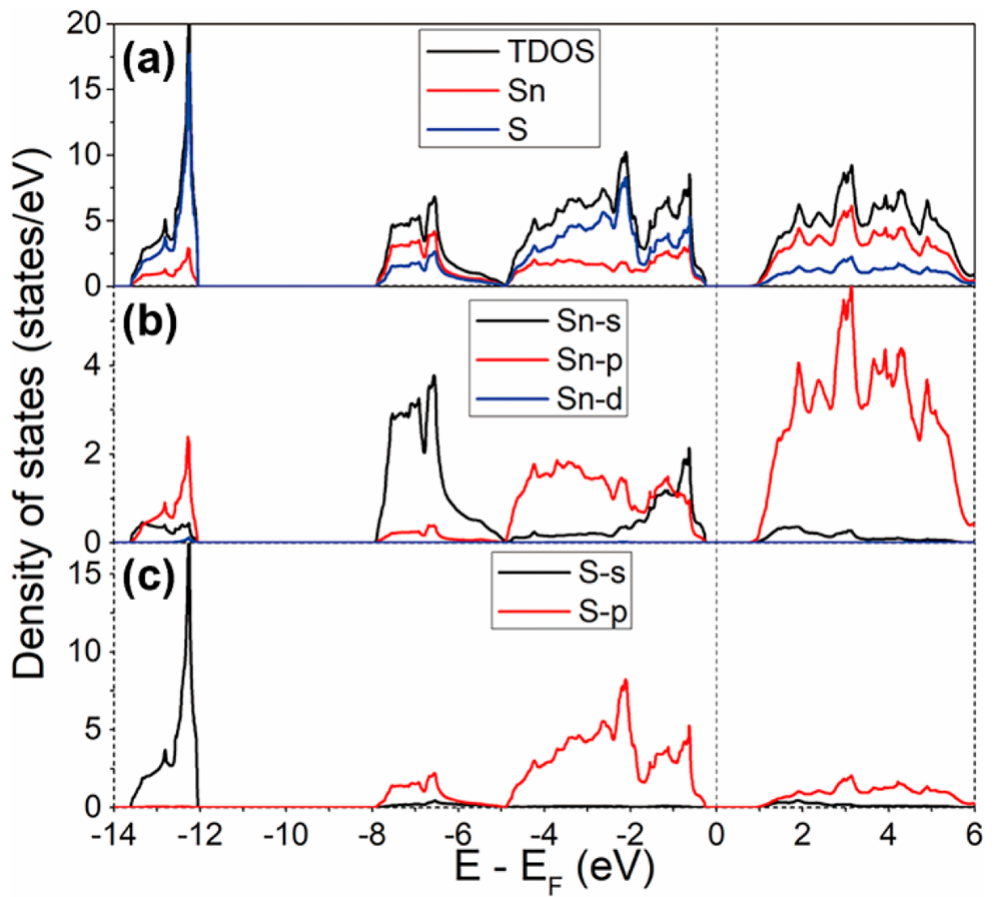
Calculated electronic structure of bulk SnS. Panel a shows the total density of states (TDoS), Panel b shows the projection of the electronic states onto the valence Sn s, p and d orbitals, and Panel c shows the projection onto the valence S s and p orbitals. The energy zero is set to the calculated Fermi energy *E*_F_, indicated by the dashed lines.

The calculated total density of states (TDoS) and atom-projected (partial) density of states (PDoS) for SnS are shown in Figure 2. The calculated band gap of 0.99 eV is in agreement with other theoretical studies,^10,47^ and is close to the experimental value of 1.07 eV for bulk SnS.^12^ As previously reported,^47^ the DoS near the valence-band edge is primarily composed of Sn 5s/5p states and S 3p states, whereas the conduction band is formed from Sn 5p and S 3p states. The Fermi level is close to the valence-band edge, implying that SnS is a p-type semiconductor, again as found experimentally.^11,18^

**Figure 3:**
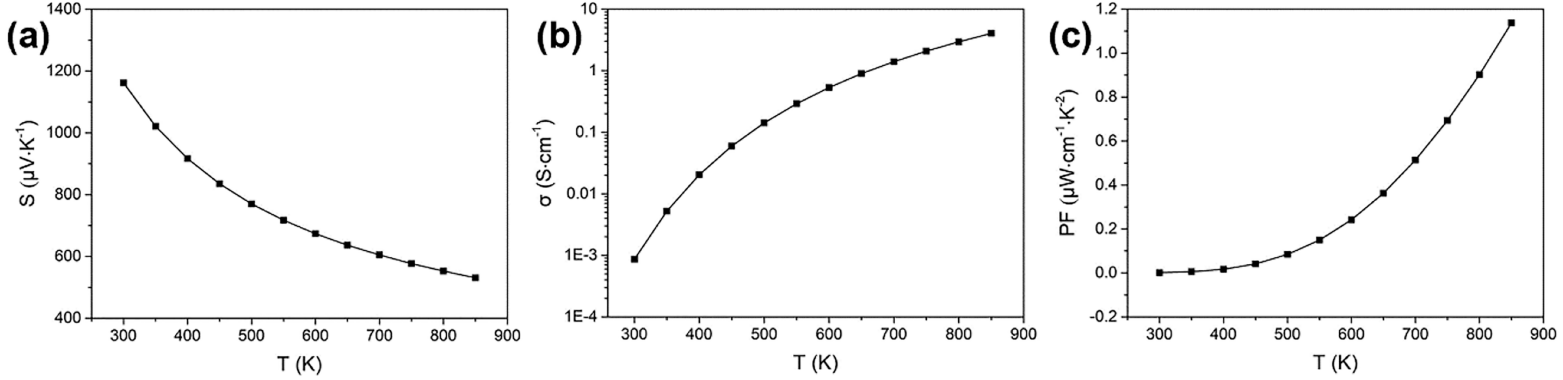
Temperature-dependent electrical transport properties of SnS calculated under the constant relaxation-time approximation with a fixed relaxation time τ = 1.51 × 10^–15^ s:^41^ (a) Seebeck coefficients (*S*), (b) electrical conductivity (σ), and (c) power factor (PF, *S*^2^σ).

The temperature-dependent electrical conductivity (σ), Seebeck coefficient (*S*), and power factor (PF, *S*^2^σ) of SnS, calculated under the constant relaxation-time approximation with a fixed relaxation time τ = 1.51 × 10^–15^ s,^41^ are shown in Figure 3. The positive Seebeck coefficients confirm that SnS is a p-type semiconductor, which is consistent with the position of the Fermi level in the calculated DoS. The Seebeck coefficient decreases and the electrical conductivity increases with increasing temperature, as a result of which the highest PF of 1.13 μW·cm^–1^·K^–2^ is obtained at 850 K. Our calculated values are again consistent with those from previous theoretical studies,^41^ and will be discussed below, alongside our experimental measurements for SnS thin films.

### 3.2 Thermal Decomposition Analysis of [Sn(C_4_H_9_)_2_(S_2_CN(C_2_H_5_)_2_)_2_]

**Figure 4:**
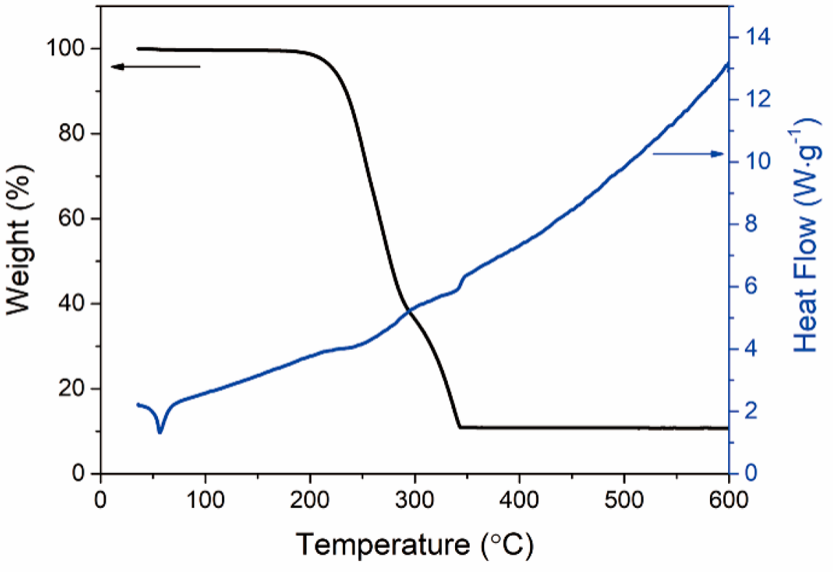
TGA (black) and DSC (blue) data for [Sn(C_4_H_9_)_2_(S_2_CN(C_2_H_5_)_2_)_2_] as a function of temperature.

The thermal decomposition of the [Sn(C_4_H_9_)_2_(S_2_CN(C_2_H_5_)_2_)_2_] precursor was examined by TGA and DSC (Figure 4). The first loss of weight begins around 180 °C and is finished by 286 °C (approximately 58% weight loss); the second decomposition step occurs between 287 and 345 °C (a weight loss of 31%). The remaining residue (11%) of the original [Sn(C_4_H_9_)_2_(S_2_CN(C_2_H_5_)_2_)_2_] complex is lower than the theoretical percentage for SnS (29%) possibly due to sublimation of SnS.^29^ The DSC data [Sn(C_4_H_9_)_2_(S_2_CN(C_2_H_5_)_2_)_2_] shows a sharp endothermic peak near 50 °C, corresponding to melting of the complex. The two broad endothermic peaks between 180–350 °C can be attributed to the decomposition steps, which is consistent with the TGA data. There is no significant weight loss above 350 °C but the increasingly upward trend of the DSC curve suggests continued crystallization of SnS.^48^ Thus, a deposition temperature above 350 °C is required to ensure complete decomposition of the precursor for SnS film preparation, and subsequent high quality crystal growth. The decomposition pathway and thermal analysis data for [Sn(C_4_H_9_)_2_(S_2_CN(C_2_H_5_)_2_)_2_] are in good agreement with earlier studies.^49,50^

### 3.3 Effects of AACVD Processing Parameters on SnS Films

The solvent selected for use in this work, toluene, has much higher heat of combustion (3910 kJ·mol^–1^) and vaporization (38 kJ·mol^–1^) than most other common solvents; it evaporates slowly allowing heterogeneous nucleation, even above 400 °C.^51^ The performance of the films obtained by AACVD is directly associated with the film structure,^52^ as the preferred orientation, density, crystallinity, and particle size and shape, depending on deposition conditions, could influence electron transport. Here, we examine the growth of the SnS films deposited under different deposition conditions focusing on the effects of carrier gas flow rate, solution concentration, and deposition temperature on the composition, microstructure, and electrical properties of the resulting SnS films.

#### 3.3.1 Growth of the SnS Films in the Hot Wall Reactor

SnS films were deposited on sets of three 2.5 × 1.5 cm glass substrates lined up end-to-end in a Carbolite tube furnace, which acts as a hot wall reactor for the CVD. In order to explore the growth of SnS films as a function of distance along the hot zone of the reactor, we first determined deposition temperatures from thermal profiles for different “set” temperatures (shown in Figure S1). Following trial experiments, we commenced with a flow rate of 150 cm^3^·min^–1^, a precursor solution concentration of 0.04 mol·L^–1^, and a furnace (set) temperature of 450 °C. Microstructures of the resulting films (Figure S5) showed that effective film deposition occurred at 5–9 cm from the tube inlet where the actual deposition temperatures increased from approximately 355–423 °C. Detailed structural and microstructural characterization of these films (Figures S5–S7 and Table S1–S3), revealed that all the films were single-phase orthorhombic SnS (JCPDS card No. 01-075-1803). The change of morphology along the tube (Figure S5) results from a combination of effects, including the change in deposition temperature and reduction in reactant-gas concentration with gas flow down the tube.^52^ The degree of preferred orientation was evaluated in terms of the Logtering factor (details in the Supporting Information). The films in the central region (380–412 °C) exhibit similar morphology of connected sheets with similar texturing (Table S3), reflecting compensation between changing deposition temperature and reactant-gas concentration. Consequently, the room temperature electrical conductivities of films from the central region are similar (Figure S7), approximately 0.02 S·cm^–1^, which is comparable to reported values for SnS films prepared by AACVD and spin coating.^25,26^

#### 3.3.2 Effects of Solution Concentration and Flow Rate

While keeping constant both the furnace temperature (450 °C) and carrier gas flow rate (150 cm^3^·min^–1^), the effects of solution concentration (0.02, 0.04, and 0.06 mol·L^–1^) on the quality of the deposited SnS films were investigated; the deposition temperature was 380 °C. The XRD, microstructure and electrical conductivity data (Figures S8–S10 and Tables S4–S6) show that single phase SnS films containing bundles of sheets were successfully prepared, which predominantly exhibit (1 *k* 1) preferred orientation with respect to the substrate. With increasing solution concentration, the film thickness and the degree of preferred orientation in the (1 *k* 1) plane increased. The SnS films deposited using a solution concentration of 0.04 mol·L^–1^ exhibit the highest electrical conductivity of ∼0.02 S·cm^–1^.

Having optimized the solution concentration (0.4 mol·L^–1^) and retained the same furnace temperature (450 °C), SnS films were also prepared using a flow rate of 300 cm^3^·min^–1^; this resulted in an optimum deposition temperature of 442 °C. As the flow rate increased from 150 to 300 cm^3^·min^–1^, the film thickness decreased from ∼7 μm to ∼4.4 μm and the microstructure became less compact (Figure S9), leading to a decrease of electrical conductivity to ∼0.0015 S·cm^–1^ (Figure S10). Thus, as noted above, SnS films deposited using a solution concentration of 0.04 mol·L^–1^ and the lower flow rate of 150 cm^3^·min^–1^ exhibit the highest electrical conductivity of ∼0.02 S·cm^–1^.

#### 3.3.3 Effects of Deposition Temperature

**Figure 5:**
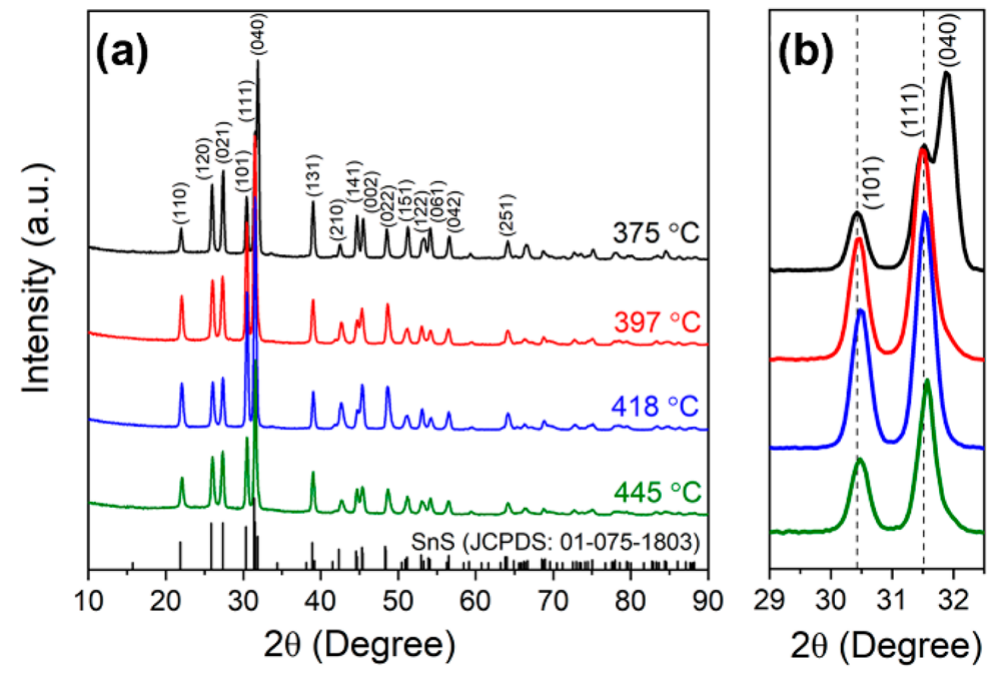
(a) XRD patterns for SnS films deposited at 375 to 445 °C; (b) magnified region from part a.

Having optimized the solution concentration and flow rate (0.4 mol·L^–1^ and 150 cm^3^·min^–1^ respectively) on the basis of microstructure and the highest electrical conductivity, we focused on the effects of deposition temperature. Good quality SnS films were produced with deposition zone temperatures of 375, 397, 418, and 445 °C (from set temperatures of 400, 450, 500, and 550 °C in Figure S1). XRD patterns for the SnS films deposited at different temperature are presented in Figure 5. The reflections can be indexed to an orthorhombic structure (space group *Pbnm*) SnS (JCPDS No. 01-075-1803), with no secondary peaks, confirming monophasic SnS. The XRD patterns for the SnS films deposited at 375 °C exhibit a strong (0 4 0) peak, indicating anisotropy; in contrast, for films deposited at 397–445 °C, the (1 1 1) peak at 31.5° is strongest. There is also a slight shift of (1 1 1) and (1 0 1) peaks to higher angles with increasing deposition temperature (Figure 5b) possibly due to the decrease of lattice parameters. The (1 1 1) and (1 0 1) peaks of the SnS film deposited at 445 °C are broadened, which may result from the smaller crystallite size. Refined lattice parameters by Rietveld method for the SnS films are presented in Table 1. With increasing deposition temperature lattice parameters *a* and *c* decrease slightly, while parameter *b* increases slightly; nevertheless the cell volumes decrease slightly, supporting the proposal above for the effects of lattice shrinkage.

**Table 1:** Refined Lattice Parameters, Cell Volumes, and Refinement Factors for SnS Films Deposited at 375 to 445 °C by Rietveld Refinement

	lattice parameters (Å)						
deposition temperature (°C)	*a*	*b*	*c*	cell volume (Å^3^)	crystallite size (nm)	*R*_wp_	GOF
375	4.3231(3)	11.2043(7)	3.9883(3)	193.18(2)	170(40)	6.87	1.84
397	4.3035(4)	11.2080(11)	3.9975(3)	192.81(3)	150(17)	7.39	1.83
418	4.3063(3)	11.2083(12)	3.9960(3)	192.87(3)	116(30)	9.66	2.09
445	4.3024(4)	11.2272(11)	3.9963(4)	193.01(3)	100(20)	9.25	1.98

**Figure 6:**
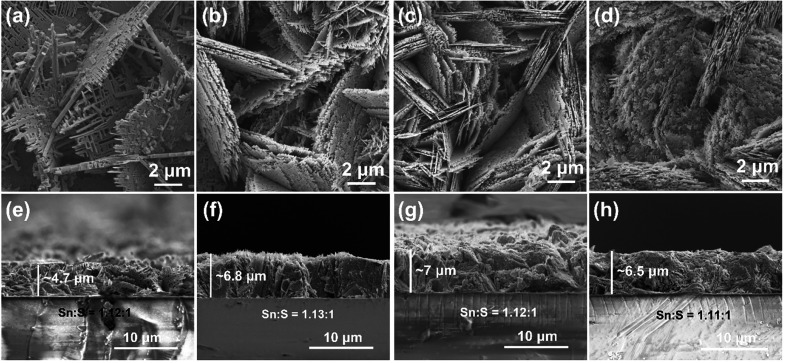
SEM micrographs, cross-sectional images, and Sn:S ratio (as measured by EDX spectroscopy) for SnS films deposited using a flow rate of 150 cm^3^·min^–1^ and a solution concentration of 0.04 mol·L^–1^ at temperatures of 375 °C (a, e), 397 °C (b, f), 418 °C (c, g), and 445 °C (d, h).

SEM micrographs and cross sectional images of the SnS films deposited at 375 to 445 °C are presented in Figure 6. As the deposition temperature increases, the form of the grains changes from thick porous sheets to clusters of thin sheets, and the films become more dense and compact with better coverage. There is significantly less porosity in the SnS films deposited at the higher temperatures of 418 and 445 °C (Figure 6c,d,g,h). This could lead to an increase in carrier mobility and concentration, which is beneficial for electrical conductivity.^25^ As the Seebeck coefficients and electrical conductivity are inversely related with respect to carrier concentration, the Seebeck coefficients might be reduced.^53^ Indeed, the higher deposition temperatures enable faster decomposition reactions as the transported material reaches the substrate surface more quickly without extended transport by diffusion.^52,54^ With increasing deposition temperature, the film thickness increases from ∼4.7 μm to ∼7 μm (Figure 6), while crystallite size decreases (Table 1). The obvious increase in thickness of the thin films is almost certainly due to the higher deposition rate at higher deposition temperatures. The decomposition reactions tend to be faster at higher temperatures, so the transported precursor reacts on the surface at a faster rate. There is minimal variation in composition on the basis of Sn:S ratios (Figure 6 and Table S7). On the basis of the TGA results (Figure 4) the decomposition of the precursor [Sn(C_4_H_9_)_2_(S_2_CN(C_2_H_5_)_2_)_2_] is completed around 350 °C and there is no significant weight loss above 350 °C, suggesting that the decomposition product is SnS above 350 °C without obvious further sublimation of Sn or S. Thus, irrespective of deposition temperature there are only slight differences in composition and stoichiometry.

As XRD data (Figure 5) and the microstructures (Figure 6) indicated texturing in the films, Lotgering factors were determined (Table 2); all values are in the range 0.14 to 0.22 suggesting modest texturing. While films deposited at 375 °C have more preferred orientation (0 4 0) compared to SnS films deposited at higher temperatures, there is a general trend of increasing Lotgering factor with increasing temperature; the film deposited at 418 °C exhibiting the highest values. The SnS films deposited at 397–445 °C exhibit dominant (1 *k* 1) orientation, which indicates the plate-like grains are aligned in the *ac* plane and stack in the *b* axis direction. Such features are visible in Figure 6a–c.

**Table 2:** Lotgering Factors for SnS Films as a Function of Deposition Temperature

deposition temperature (°C)	preferred orientation	Lotgering factor (±10%)
375	(0 4 0)	0.149
397	(1 *k* 1)	0.171
418	(1 *k* 1)	0.219
445	(1 *k* 1)	0.179

**Figure 7:**
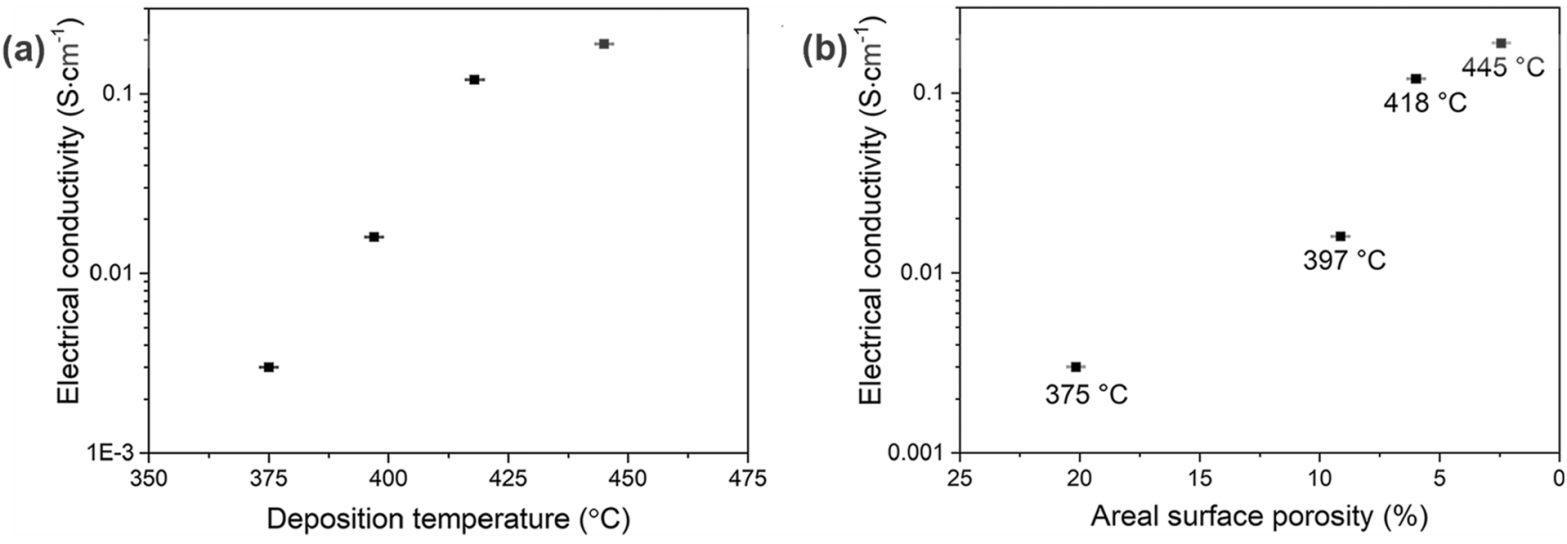
(a) Room-temperature electrical conductivity for SnS films as a function of deposition temperature. (b) Room-temperature electrical conductivity for SnS films as a function of areal surface porosity. Uncertainty bars represent 1% and are comparable in size with data points.

To validate the quality of the films, we first determined the electrical conductivity from sheet resistance at room temperature (Figure 7), prior to detailed characterization. The relationship between electrical conductivity and porosity of the films is shown in Figure 7b. With increasing deposition temperature, the electrical conductivity increased significantly from 0.003 S·cm^–1^ to 0.19 S·cm^–1^ (more than 60× increase) as a result of the improvement in microstructure reflected in the improved film coverage, denser structure (Figure 6), and reduced porosity (Figure 7b). Thus, all films exhibited good electrical conductivity, with those from the higher deposition temperatures being exceptionally good for SnS.^54,55^ The SnS films deposited at 445 °C with the highest electrical conductivity were annealed at 445 °C under nitrogen for 2 h to assess the effects of annealing (Figure S12, Figure S13, and Table S8). The SEM images (Figure S13), show that the grains in the annealed samples are wider and better connected to each other than in the as-prepared samples. The results in Table S8 indicate that the electrical conductivity of the annealed samples is ∼3 times larger than that of unannealed samples. Therefore, annealing provides a route for enhancement of thermoelectric performance.

### 3.4 TEM Analysis

**Figure 8:**
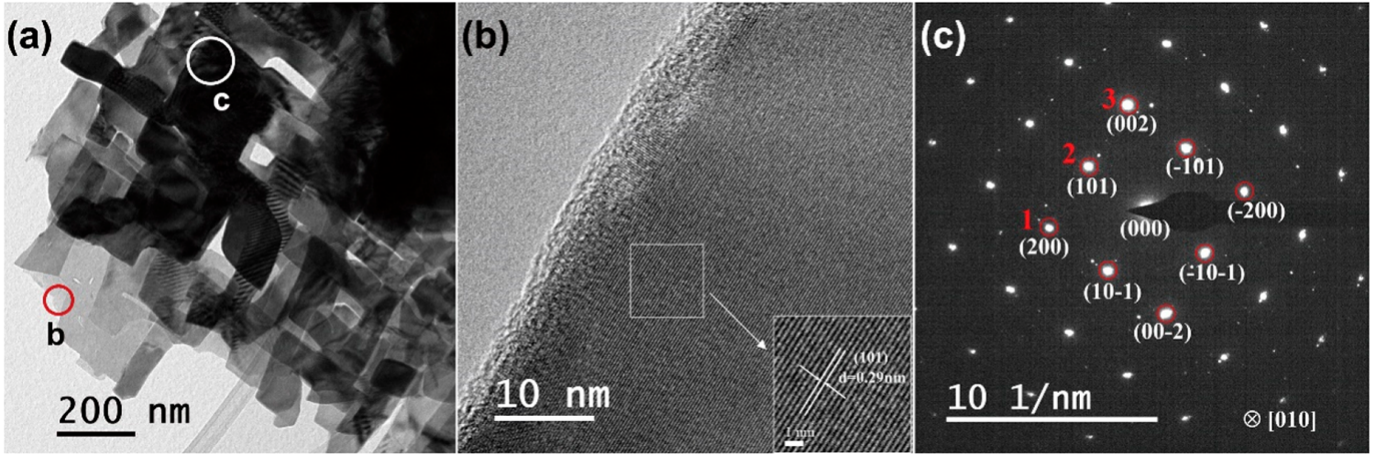
(a) Low-magnification bright-field TEM image for an SnS film deposited at 445 °C, (b) HRTEM image taken from the red region in part a, and (c) selected area electron diffraction pattern taken from the region identified by the white region in part a.

Figure 8a shows a low-magnification bright-field TEM image for flakes extracted from an SnS film deposited at 445 °C; the image is dominated by stacked, rectangular thin nanocrystals which are several hundred nanometers in size, consistent with the SEM image (Figure 6d). The HRTEM image (Figure 8b) of the red region in Figure 8a confirms the interplanar spacing of ∼0.29 nm, which matches the (101) crystal plane of orthorhombic SnS (JCPDS card No. 01-075-1803). Figure 8c shows the SAED pattern collected along the [010] zone axis. The bright dots in the SAED pattern indicate the high crystallinity of the SnS film deposited by AACVD. The interplanar spacings of the diffraction spots 1, 2, and 3 shown in Figure 8c are approximately 0.211, 0.290, and 0.201 nm, corresponding well to the planes of (2 0 0), (1 0 1), and (0 0 2) respectively. The angles between (2 0 0) and (1 0 1), and between (2 0 0) and (0 0 2) are approximately 46° and 90°; consistent with the XRD analysis and earlier work.^56^ The small white spots near the bright spots appear to belong to the other SnS crystals beneath the main crystal in area c of Figure 8a.

**Figure 9:**
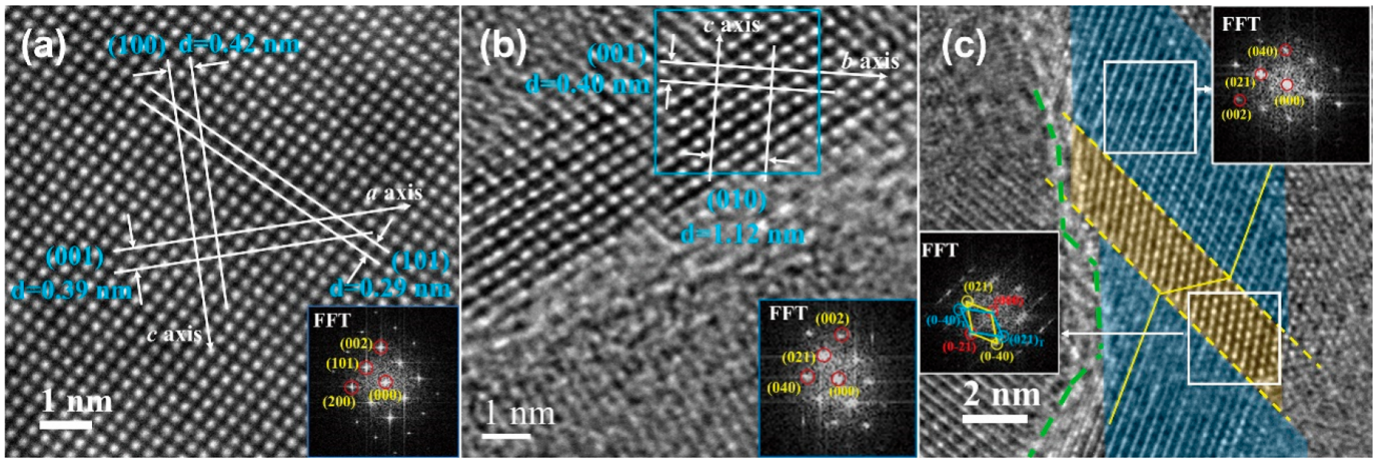
(a) HRTEM image along the [010] zone axis, (b) HRTEM image along the [100] zone axis, and (c) HRTEM image of a twined structure for the SnS film deposited at 445 °C. FFT images are shown as insets. The yellow dash lines in part c depict a twin boundary (which were observed occasionally in SnS films), and the green dash line in the image denotes a grain boundary.

Parts a and b of Figure 9 show HRTEM images along the [010] zone axis and [100] zone axis, respectively, and the associated FFT patterns. The latter match well the orthorhombic SnS structure (JCPDS card No. 01-075-1803) and are in accordance with earlier TEM work.^56,57^ The interplane spacings of (1 0 0), (0 1 0), and (0 0 1) are estimated to be 0.42, 1.12, and 0.39 nm, agreeing with lattice parameters for *a*, *b*, and *c* from the XRD refinement (Table 1). In SnS films deposited at 445 °C twinned structures were occasionally observed (examples in Figure 9c and Figure S14); the twin boundaries are identified by yellow dash lines. Yu et al. demonstrated that twin-boundary scattering can lead to a reduction of lattice thermal conductivity, which is beneficial to thermoelectric performance.^58^ In Figure 9c, a grain boundary is highlighted by the green dash line.

### 3.5 Raman Spectroscopy and Optical Bandgap

**Figure 10:**
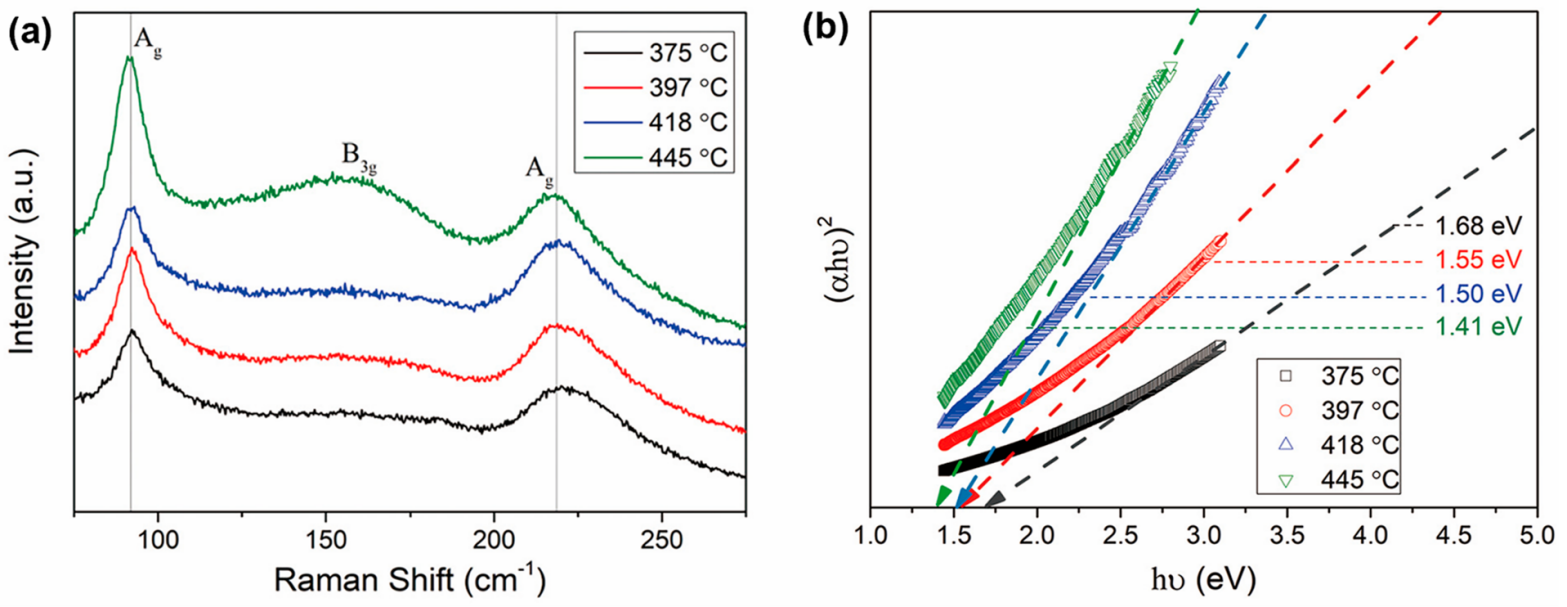
(a) Raman spectra and (b) calculated optical band gaps for SnS films deposited at 375, 397, 418, and 445 °C.

Raman spectra for SnS films deposited at different temperature are presented in Figure 10a. The peaks located at 92 cm^–1^and 219 cm^–1^ and a broad peak at 161.6 cm^–1^ agree well with the Raman spectra reported for SnS films prepared by AACVD and spin coating.^25,57^ The Raman peaks at 92 and 219 cm^–1^ are attributed to the A_g_ phonon mode and the peak at 162 cm^–1^ originates from the B_3g_ mode.^59^ There is no significant movement of the Raman peaks with increasing deposition temperature.

The optical band gap energies of the SnS films were estimated from absorption data using the following equation:^60^
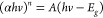
2Here α is the absorption coefficient, *hν* is the photo energy, *A* is a constant, and *E_g_* is the bandgap. In the present work, equation is satisfied for *n* = 2, implying a direct allowed transition in these SnS films. By plotting the curves of (α*hν*)^2^ vs *hν* and extrapolating the linear region (Figure 10b), the direct bandgaps of the SnS films reduce from ∼1.68 eV to ∼1.41 eV with increasing deposition temperature. The decrease of the bandgap with temperature and the direct band nature for the SnS films is consistent with previous findings for SnS films.^25,61,62^ These bandgap values are close to the values reported by Kevin et al.^29^ and Ramasamy et al.^25^

### 3.6 Electrical Transport Properties of SnS Films

**Figure 11:**
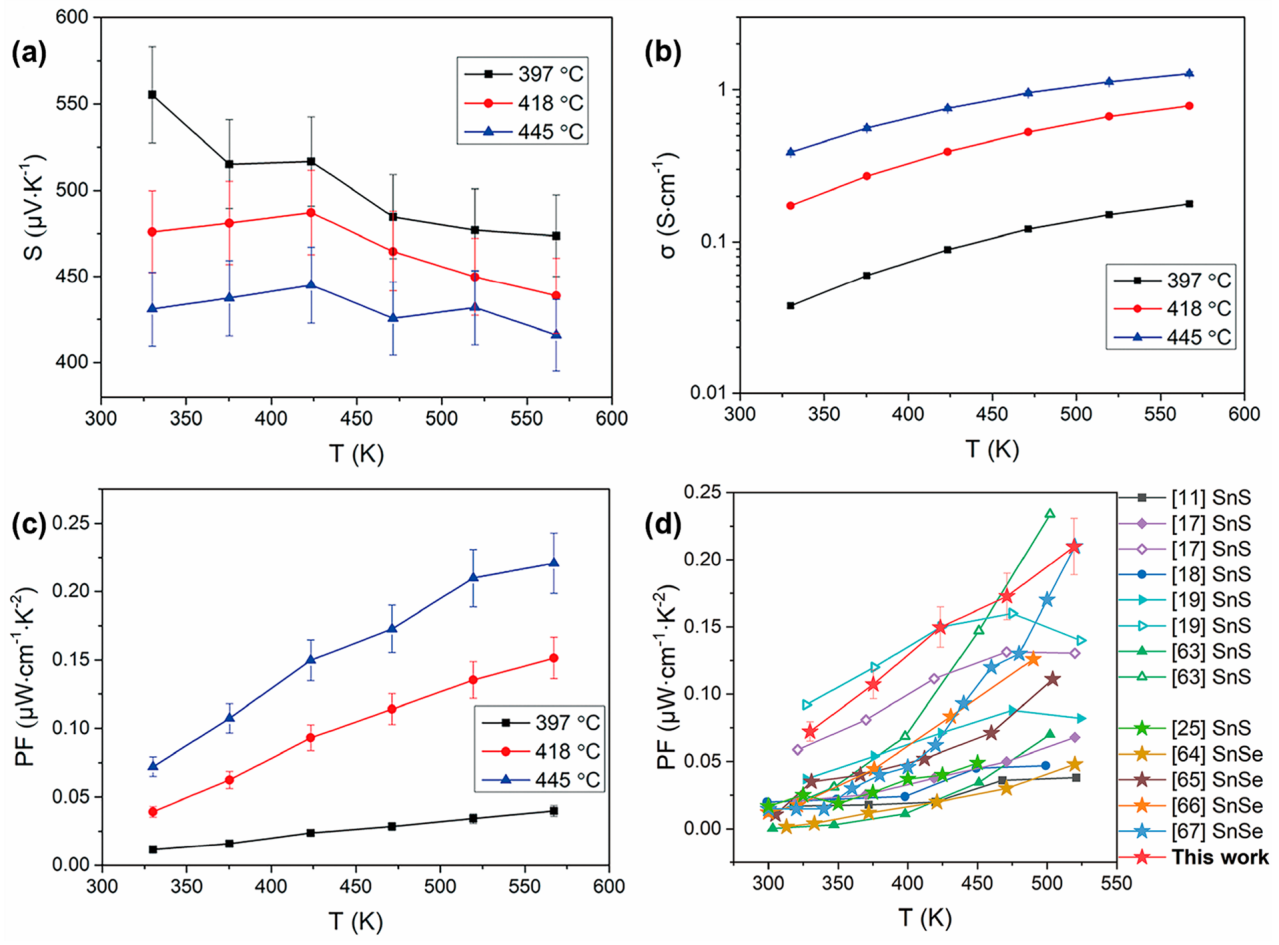
Temperature dependence of (a) Seebeck coefficient (*S*), (b) electrical conductivity (σ), and (c) power factor (PF) for SnS films deposited at 397, 418, and 445 °C; (d) Comparison of the PF in this work (red stars) with reported values for SnS bulk materials with similar grain sizes to that of our SnS films and SnSe and SnS thin films (star symbols) prepared by low-cost deposition techniques.^11,17−19,22,63−67^

The temperature dependence of the electrical transport properties of the SnS films deposited at 397–445 °C are presented in Figure 11. Data for films deposited at 375 °C have been omitted as the electrical conductivity was too low to be determined reliably. The positive Seebeck coefficient confirms the p-type nature of the thin films. It can be seen that the Seebeck coefficients decreased with increasing deposition temperature (Figure 11a), and the film deposited at 397 °C exhibits the largest Seebeck coefficient of 556 μV·K^–1^ at 330 K. As expected, electrical conductivity increased significantly with increasing deposition temperature (Figure 11b) possibly due to the increased carrier mobility and concentration caused by the denser structure and better coverage (Figure 6).^25^ The highest electrical conductivity around 1.3 S·cm^–1^ at 570 K was obtained for the SnS film deposited at 445 °C. This is comparable to electrical conductivities reported for a number of SnS bulk samples at ∼600 K,^12,63^ and even higher than reported values for many SnS bulk ceramics.^11,15,17,18^ From parts a and b of Figure 11, the increase of electrical conductivity for the SnS films prepared at 418 and 445 °C below 425 K is inferred to result from the increase of carrier mobility since an increase of carrier concentration would cause a reduction of the Seebeck coefficient.^63^Figure 11c shows the temperature-dependent power factor data for the SnS films; a maximum value of 0.22 μW·cm^–1^·K^–2^ at 570 K was achieved for films deposited at 445 °C. It is noted that the values and the trends for σ, *S*, and PF at temperatures in the range 300–450 K for our SnS film deposited at 397 °C (lowest deposition temperature in Figure 11) are comparable with the results of Robinson et al. for SnS film prepared by LPCVD.^22^ The maximum PF value for our SnS films deposited at 445 °C (with PF reaching 0.22 μW·cm^–1^·K^–2^ at 570 K) are significantly higher than the reported value.^22^ The exceptional performance achieved here reflects the high quality of the films (confirmed by TEM and Raman measurements, Figures 8–10) and the dense homogeneous microstructure (Figure 6).

Figure 11d shows a comparison of the PF data from this work with reported values for polycrystalline SnS bulks with similar grain size to that of our SnS film and SnSe and SnS thin films deposited by low-cost techniques including spin coating, electrodeposition, thermal evaporation, and pulsed laser deposition.^11,17−19,22,63−67^ The PF values for our SnS film deposited at 445 °C by AACVD in the temperature range of 300–520 K are at least comparable to, and in most cases higher than, the PF values reported for SnS bulks with similar grain size with the SnS film. Indeed, up to 520 K, our PF data exceeds that for all SnSe films deposited by other low-cost techniques (Figure 11d). While SnS has a similar structure to SnSe, sulfur has the advantage of being much more abundant and less expensive than Se.^68^ Thus, our results indicate that low-cost, environment-friendly SnS films are promising candidates for thermoelectric applications, and further, the AACVD technique is a flexible, easily operated, and dependable method for the preparation of high-quality films for thermoelectric applications.

Comparing the experimental results in Figure 11 to the calculations for bulk SnS in Figure 3 shows similar trends and generally good agreement. The experimental maximum electrical conductivity of 1.3 S·cm^–1^ at 570 K is somewhat higher than calculated ∼0.5 S·cm^–1^ at 600 K, but the measured PF of ∼0.22 μW·cm^–1^·K^–2^ at 570 K (for films deposited at 445 °C) is close to the predicted value ∼0.24 μW·cm^–1^·K^–2^ at 600 K. We therefore consider there to be sufficient agreement between the calculations and experimental results to have confidence in the modeling. This being the case, we note that the highest predicted PF of 1.13 μW·cm^–1^·K^–2^ at 850 K is much higher than the present data for films, albeit at lower temperatures, and we take this as an indication of what could be possible with high quality SnS. Indeed, we found that the predicted PF values reported in this work are remarkably close to the PFs reported for SnS bulk ceramics (Figure S16),^11,17,18,63^ which indicates both that the calculated results are reliable and that the experimental results on SnS films are highly encouraging.

### 3.7 Thermal Conductivity and *zT* of SnS Films

**Figure 12:**
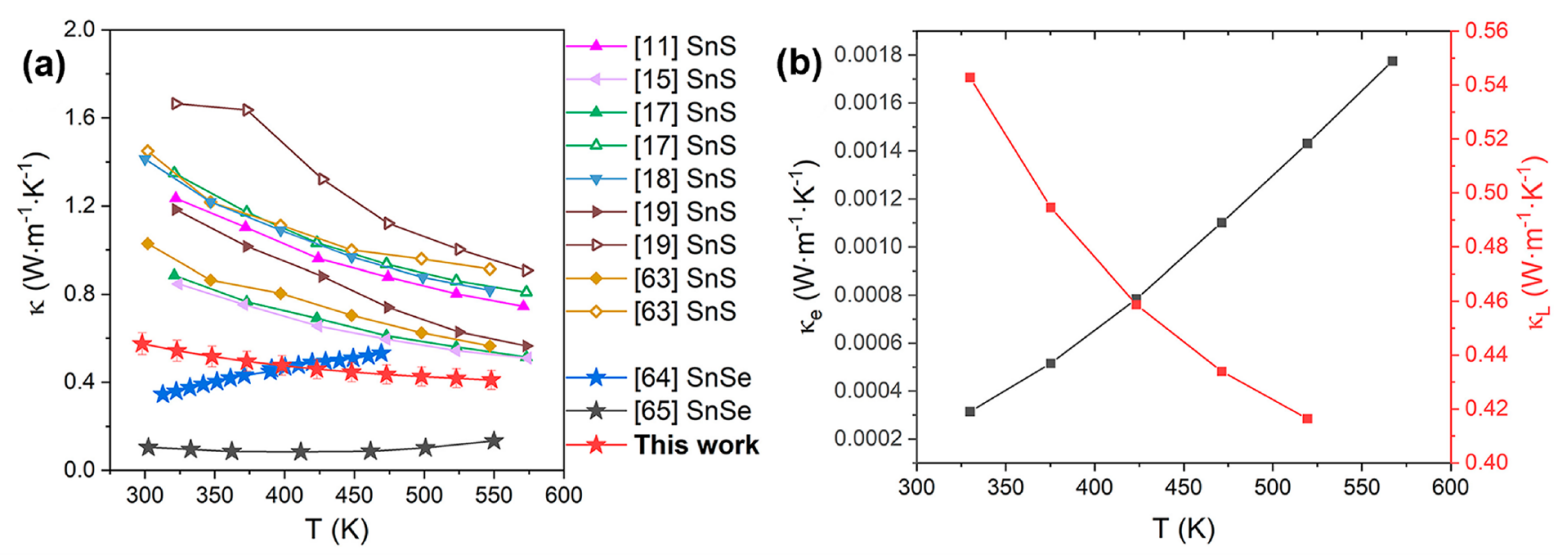
(a) Temperature dependence of thermal conductivity for SnS films deposited at 445 °C and comparison of thermal conductivity in this work (red stars) with reported values for SnS bulks with similar grain size to that of our SnS films, and SnSe and SnS thin films (star symbols) prepared by low-cost deposition techniques.^11,15,17−19,63−65^ (b) Temperature dependence of electronic thermal conductivity (*κ*_*e*_) and lattice thermal conductivity (*κ*_*L*_) for SnS films deposited at 445 °C.

The temperature-dependence of thermal conductivity at 300–550 K of the SnS film deposited at 445 °C obtained by TFA measurement are shown in Figure 12a. As temperature increases, the thermal conductivity of the SnS film decreases from ∼0.57 to ∼0.41 W·m^–1^·K^–1^. The reduction of the thermal conductivity for the SnS film with increasing temperature is consistent with the normal trends in the thermal conductivity of SnS bulk ceramics reported in the literature (Figure 12a).^11,15,17−19,63−65^ The thermal conductivity for the SnS film in our work is lower than all reported values for SnS bulk materials with a similar grain size, and is comparable to the values for SnSe films deposited by electrodeposition.^64^ The electronic thermal conductivity (*κ*_*e*_) is calculated approximately via the Wiedemann–Franz law^63^*κ*_*e*_ = *LσT*, where *L* is Lorenz number (2.45 × 10^–8^ W·Ω·K^–2^). The lattice thermal conductivity (*κ*_*L*_) can then be calculated as *κ*_*L*_ = κ – *κ*_*e*_.^63^ In our previous work, the lattice thermal conductivity of SnS was calculated,^44^ and we showed that the lattice thermal conductivity reduced from ∼2.15 W·m^–1^·K^–1^ at 300 K to ∼1.2 W·m^–1^·K^–1^ at 550 K, both of which are larger than the results obtained for SnS thin films (Figure 12b). Compared to the SnS perfect bulk structure employed in the calculations, the experimental SnS thin films contain a variety of defects including holes, grain boundaries and the interfaces between the film and substrate which could enhance phonon scattering and reduce thermal conductivity.

**Figure 13:**
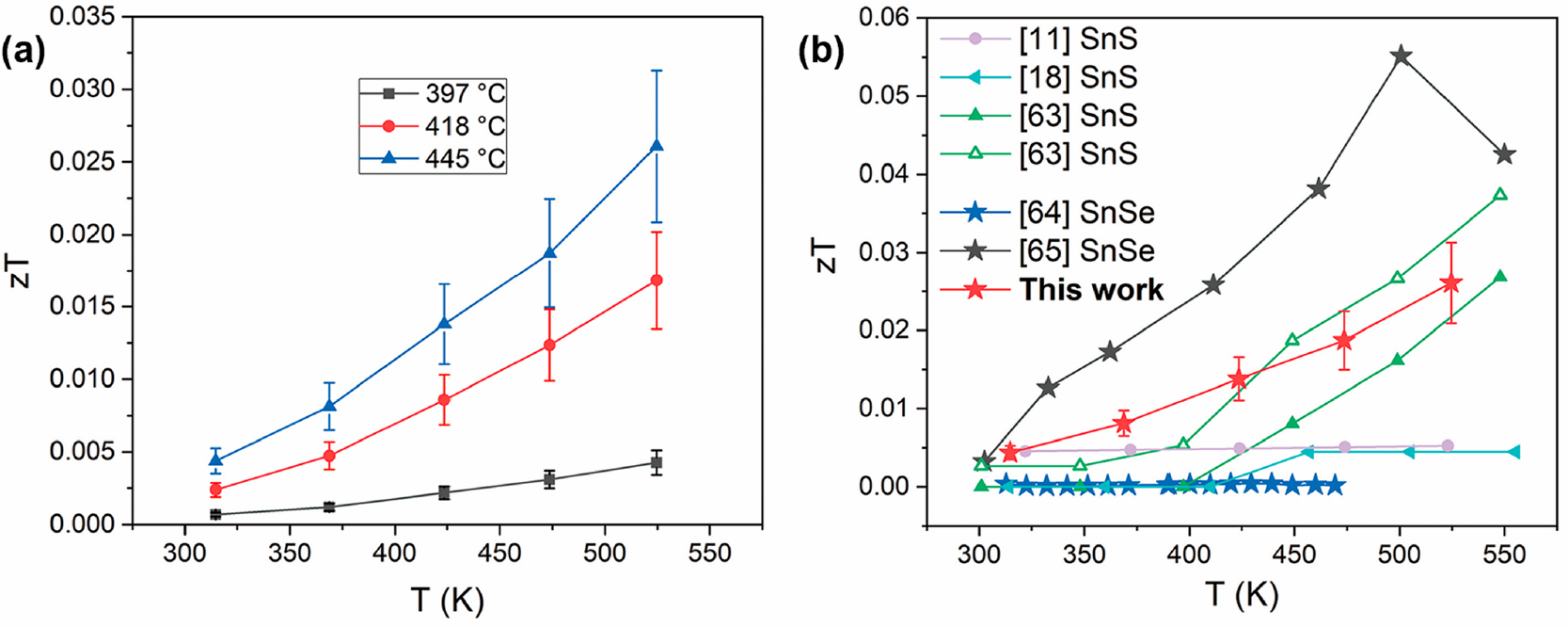
(a) Temperature dependence of *zT* for the SnS films deposited at 397, 418, and 445 °C. (b) Comparison of *zT* in this work (red stars) with reported values for SnS bulks with similar grain size to that of our SnS film, and SnSe and SnS thin films (star symbols) prepared by low-cost deposition techniques.^11,18,63−65^

The *zT* values at the temperature range of 330–520 K for the SnS films deposited at 397, 418, and 445 °C were evaluated using the data measured by ZEM-3 and TFA, and the results are shown in Figure 13a. The maximum value of *zT* reaches 0.026 at 520 K for the SnS film deposited at 445 °C. By comparison (Figure 13b), the *zT* values for our SnS film are larger than those for SnSe film deposited by electrodeposition,^64^ and comparable to and even larger than most of the results for SnS bulks with similar grain size to that of the SnS film.^11,18,63−65^ As noted previously, it is difficult to measure the thermal conductivity of thin films, and consequently, thermal conductivity data are rarely included in studies of thermoelectric thin films.^69^ To date, the thermal conductivity for SnS film has not previously been reported. Our work provides a feasible way of combining AACVD technique and TFA measurement to measure the thermal conductivity and determine *zT* for SnS films.

## 4 Conclusions

High quality SnS films were prepared by AACVD using dibutylbis(diethyldithiocarbamato)tin(IV) as the single source precursor. AACVD deposition parameters including solution concentration, flow rate and deposition temperature were optimized to maximize transport properties. SnS films deposited using a solution concentration of 0.4 mol·L^–1^, a flow rate of 150 cm^3^·min^–1^ and a deposition temperature of 445 °C exhibit the most compact microstructure and the highest room-temperature electrical conductivity of 0.19 S·cm^–1^. Deposition temperature has the greatest impact on electrical conductivity. The maximum power factor of 0.22 μW·cm^–1^·K^–2^ at 570 K was obtained for SnS films deposited at 445 °C. This is higher than most previous investigations of SnS bulks and SnS and SnSe films up to 520 K. We observed good agreement between the measured electrical transport properties and first-principles DFT calculations on bulk SnS. The SnS film deposited at 445 °C shows a minimum thermal conductivity value of ∼0.41 W·m^–1^·K^–1^ at 550 K; as a result, a maximum *zT* value of ∼0.026 is obtained at 520 K.

This work demonstrates that earth-abundant, low-cost and nontoxic SnS films are promising thermoelectrics. We for the first time report the thermal conductivity and the *zT* for SnS film, and provide a feasible method of measuring thermal conductivity for AACVD thin film samples by TFA system. The AACVD technique is a reliable and easy to use method for the preparation of thermoelectric films, allowing optimization of thermoelectric performance by adjustment of the AACVD parameters.

## Supporting Information Available

Temperature profiles for the furnace with set temperatures of 400, 450, 500, and 550 °C; schematic cross-sectional view of the membrane setup on TFA test chip; convergence tests for QE calculation; SEM micrographs, cross-sectional images, and composition in terms of Sn:S ratio for different regions of the SnS films deposited at a set temperature of 450 °C using a flow rate of 150 cm^3^·min^–1^; effects of flow rate and precursor concentration; XRD patterns, SEM images, and electrical conductivity for the annealed SnS films; HRTEM images of twin boundaries for SnS film deposited at 445 °C; temperature dependence of Seebeck coefficients, electrical conductivity, and power factor for SnS films deposited at 445 °C in heating and cooling cycles; comparison of the power factor for SnS as a function of temperature calculated by DFT in this work with experimental data reported for bulk SnS; SEM micrographs and cross sectional image for the SnS film deposited on the chip with Si_3_N_4_ membrane; and temperature dependence of thermal conductivity for SnS film deposited at 445 °C (PDF)
