# Supplementary material for: Enhanced Thermoelectric Performance of Tin(II) Sulfide Thin Films Prepared by Aerosol Assisted Chemical Vapor Deposition
Source: ACS Appl Energy Mater. 2023 Apr 3;6(8):4462–74. doi: 10.1021/acsaem.3c00608 (PMC10131136; doi:10.1021/acsaem.3c00608)
Supplement: Supplementary file 1 — ae3c00608_si_001.pdf [file ae3c00608_si_001.pdf]

Supporting Information

Enhanced Thermoelectric Performance of Tin(II) Sulfide Thin Films Prepared by Aerosol Assisted Chemical Vapor Deposition

Yu Liu [a], Paul D. McNaughter [b], Feridoon Azough [a], Xiaodong Liu [a], Jonathan M. Skelton [b], Andrey V. Kretinin [a, c], David J. Lewis [a], and Robert Freer* [a].*

[a] Department of Materials, University of Manchester, Oxford Road, Manchester, M13 9PL, UK.

[b] Department of Chemistry, University of Manchester, Oxford Road, Manchester, M13 9PL, UK.

[c] National Graphene Institute, University of Manchester, Oxford Road, Manchester, M13 9PL, UK.

*Corresponding author: Robert Freer, David J. Lewis

E-mail: Robert.Freer@manchester.ac.uk; david.lewis-4@manchester.ac.uk

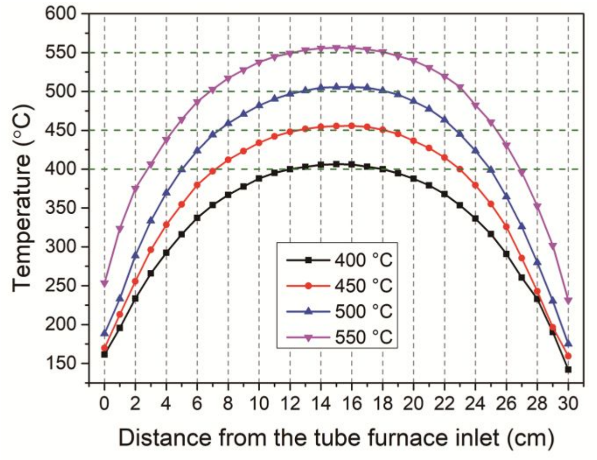

Figure S1. Temperature profiles for the furnace with set temperatures of 400, 450, 500 and 550 °C.

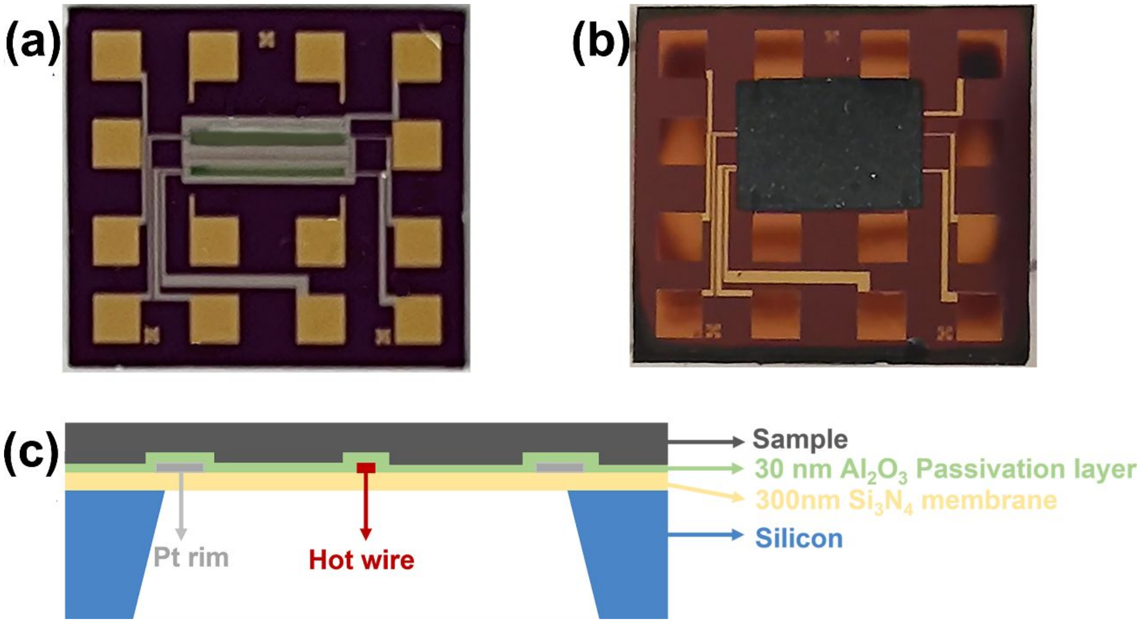

Figure S2. (a) Picture of empty test chip with the two membrane setups; (b) Picture of the SnS film deposited by AACVD on the membrane area of the test chip covered by mask; (c) Schematic cross-sectional view of the membrane setup for in-plane thermal conductivity measurement on the test chip used in this work.

A hot wire is used as a heater by applying a current. The resistivity of the wire changes with the increase of temperature, which is detected and measured. The thermal conductivity of the thin film on the test chip can be calculated from the resistivity change and the knowledge of the exact geometries of the setup.[1, 2]

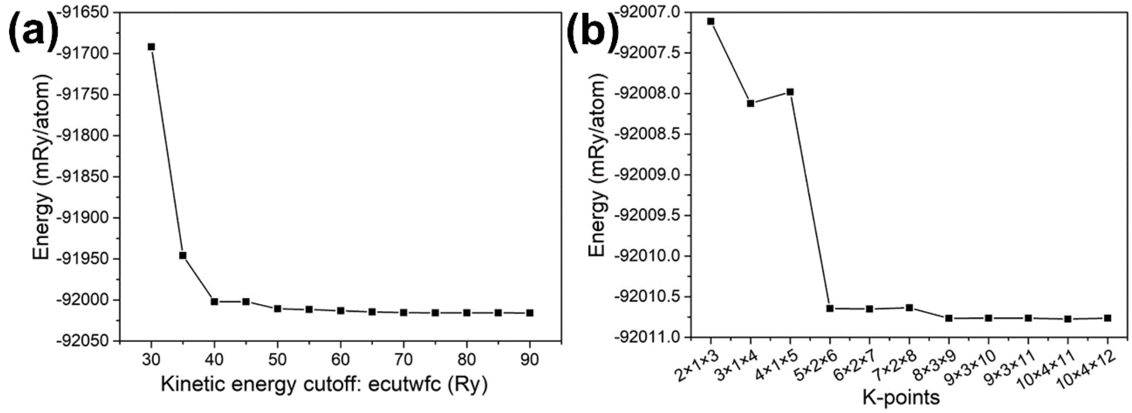

Figure S3. Convergence of the total energy of bulk orthorhombic SnS with respect to (a) the plane-wave cutoff and (b) k-point sampling.

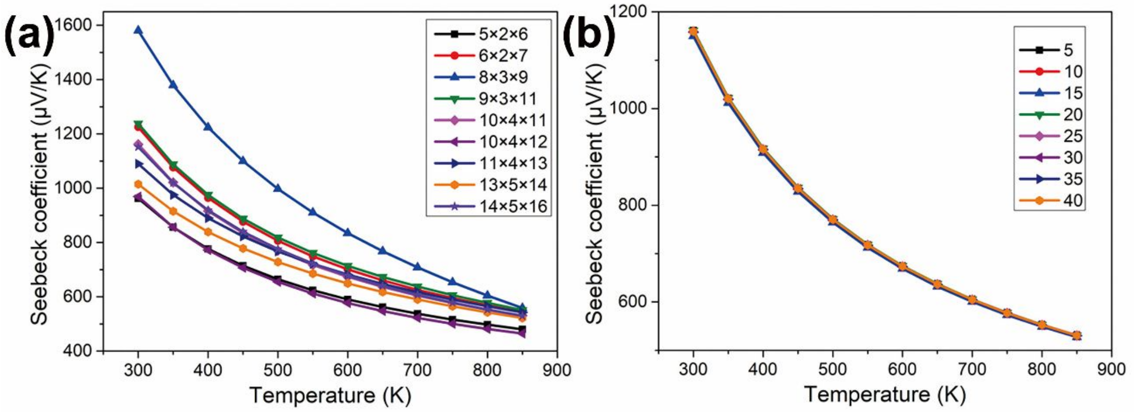

Figure S4. Calculated Seebeck coefficients for bulk orthorhombic SnS as a function of temperature obtained with (a) different *k*-point sampling grids and (b) different *lpfac*.

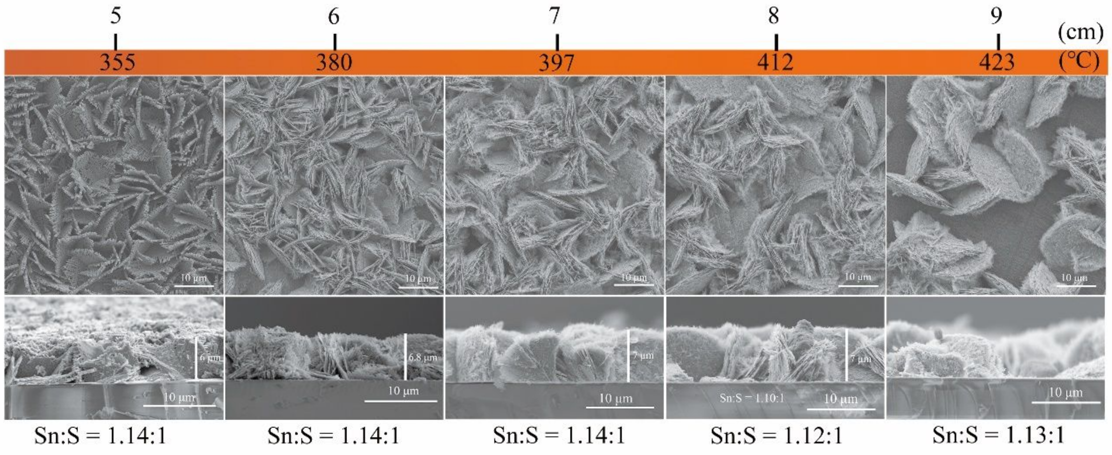

Figure S5. SEM micrographs, cross-sectional images and composition in terms of Sn:S ratio for

different regions of the SnS films deposited at a set temperature of 450 ℃ using a flow rate of 150 $cm^3 \cdot min^{-1}$. Deposition temperatures and distance from inlet tube are shown above the figure.

Table S1. EDX quantification results and Sn:S ratios for SnS film regions deposited at temperatures of 355 °C to 423 °C.

Deposition temperature (°C)	Atomic%		Sn:S
	Sn	S	
355	53.3	46.7	1.14
380	53.3	46.7	1.14
397	53.2	46.8	1.14
412	52.9	47.1	1.12
423	53.1	46.9	1.13

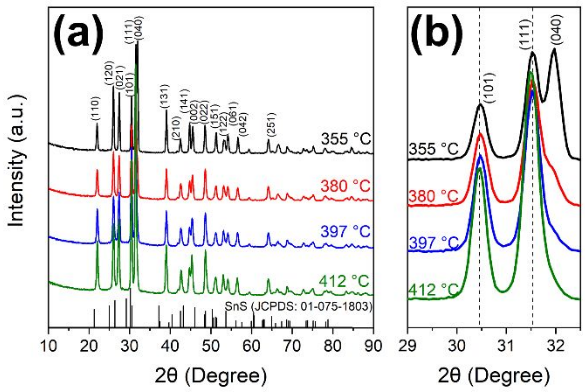

Figure S6. (a) XRD patterns for SnS films deposited at temperatures of 355 °C to 412 °C; (b) shows a magnified region from (a).

Table S2. Lattice parameters, cell volumes and refinement factors for SnS film regions deposited at different temperatures by Rietveld refinement (based on data in Figure S5).

Deposition temperature (°C)	Lattice parameters (Å)			Cell Volume (Å^3)	R_{wp}	GOF
	a	*b*	*c*			
355	4.3283(3)	11.1972(7)	3.9874(3)	193.25(2)	6.15	1.36
380	4.3202(4)	11.2075(12)	3.9906(4)	193.22(3)	6.42	1.43
397	4.3143(4)	11.2032(13)	3.9945(4)	193.07(3)	7.16	1.68
412	4.3046(3)	11.2115(11)	3.9988(3)	192.99(3)	7.42	1.83

The degree of preferred orientation of selected films was evaluated in terms of the Lotgering factor (*LF*) using XRD data and equation (S1).[3]

$$LF = \frac{p - p_0}{1 - p_0} \quad \text{(S1)}$$

where $p = \frac{\sum I\,(1\,k\,1)}{\sum I\,(h\,k\,l)}$, $p_0 = \frac{\sum I_0\,(1\,k\,1)}{\sum I_0\,(h\,k\,l)}$, $\sum I_0$ is the sum of the peak intensities of standard data, and $\sum I$ is the sum of the intensities of the measured data. *LF* = 0 corresponds to random orientation, and *LF* = 1 corresponds to perfect texture along a preferred growth direction.

Table S3. Lotgering Factors for SnS film regions prepared with different deposition temperatures (based on data in Figure S5)

Deposition temperature (°C)	Preferred orientation	Lotgering factor (±10%)
355	(0 4 0)	0.092
380	(1 k 1)	0.103
397	(1 k 1)	0.149
412	(1 k 1)	0.171

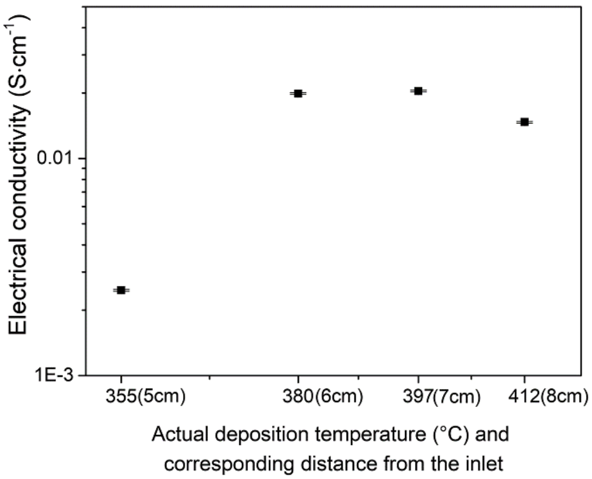

Figure S7. Room-temperature electrical conductivity for the SnS film regions as a function of deposition temperature. Distances from the tube furnace inlet are shown in brackets (see Figure S4). Uncertainty represent 1%, and are comparable in size with data points.

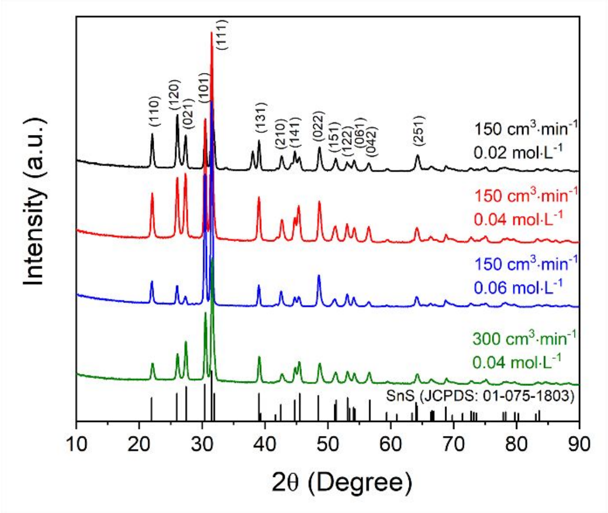

Figure S8. XRD patterns for SnS films deposited at a set temperature of 450 ℃ using different solution concentrations and flow rates; for flow rates of 150 cm^3 min^{-1} the deposition temperature was 380 ℃; for flow rates of 300 cm^3 min^{-1} the deposition temperature was 442 ℃.

Table S4. Refined lattice parameters and cell volume for SnS films deposited at a set temperature of 450 ℃ using different solution concentrations and flow rates by Rietveld refinement. For flow rates of 150 cm^3 min^{-1} the deposition temperature was 380 ℃; for flow rates of 300 cm^3 min^{-1} the deposition temperature was 442 ℃.

Flow rate ($cm^3 \cdot min^{-1}$)	Solution concentration ($mol \cdot L^{-1}$)	Lattice parameters (Å)			Cell Volume (Å^3)	R_{wp}	GOF
		a	b	c			
150	0.02	4.3093(9)	11.223(3)	3.9993(10)	193.41(8)	13.26	2.82
150	0.04	4.3041(4)	11.2097(11)	3.9986(3)	192.92(3)	7.11	1.76
150	0.06	4.3167(4)	11.2061(18)	3.9897(4)	193.00(4)	11.85	2.35
300	0.04	4.3052(5)	11.2125(13)	3.9959(4)	192.89(4)	7.90	1.71

Table S5. Lotgering Factors for SnS films deposited at a set temperature of 450 °C using different solution concentrations and flow rates. For flow rates of 150 $cm^3 min^{-1}$ the deposition temperature was 380 °C; for flow rates of 300 $cm^3 min^{-1}$ the deposition temperature was 442 °C.

Flow rate ($cm^3 \cdot min^{-1}$)	Solution concentration ($mol \cdot L^{-1}$)	Preferred orientation	Lotgering factor (±10%)
150	0.02	(1 k 1)	0.064
150	0.04	(1 k 1)	0.149
150	0.06	(1 k 1)	0.467
300	0.04	(1 k 1)	0.197

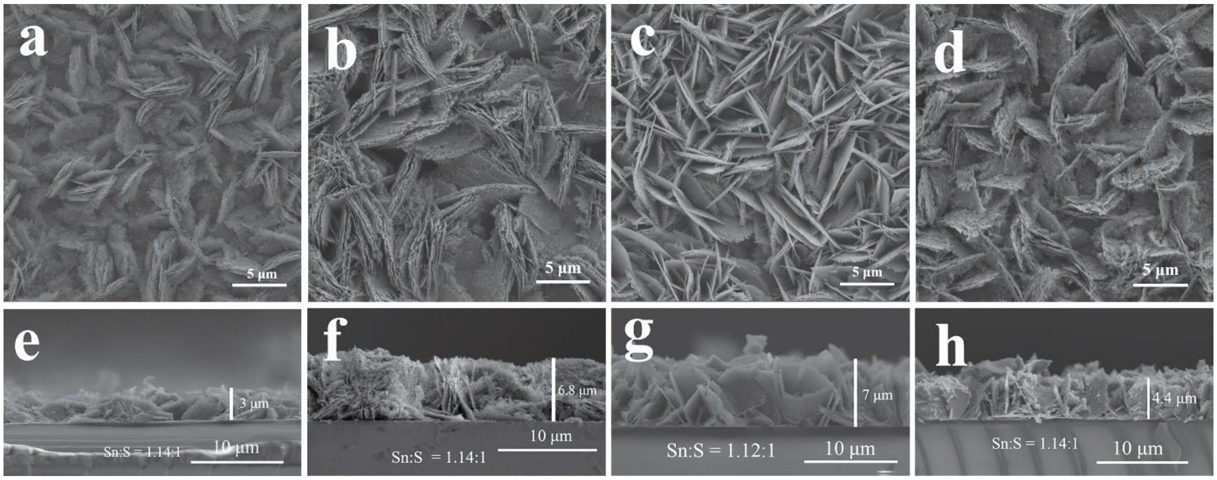

Figure S9. SEM micrographs and cross-sectional images for SnS films deposited at 380 °C using a flow rate of 150 $cm^3 \cdot min^{-1}$and solution concentrations of 0.02 $mol \cdot L^{-1}$: (a) (e), 0.04 $mol \cdot L^{-1}$ (b) (f), and 0.06 $mol \cdot L^{-1}$ (c) (g), and SnS films deposited at 442 °C using a flow rate of 300 $cm^3 \cdot min^{-1}$ and a solution concentration of 0.04 $mol \cdot L^{-1}$ (d) (h).

Table S6. EDX quantification results and Sn:S ratios for SnS films deposited at 380 ℃ using a flow rate of 150 $cm^3 \cdot min^{-1}$and solution concentrations of 0.02 $mol \cdot L^{-1}$, 0.04 $mol \cdot L^{-1}$, and 0.06 $mol \cdot L^{-1}$, and SnS films deposited at 442 ℃ using a flow rate of 300 $cm^3 \cdot min^{-1}$ and a solution concentration of 0.04 $mol \cdot L^{-1}$.

Flow rate ($cm^3 \cdot min^{-1}$)	Solution concentration ($mol \cdot L^{-1}$)	Atomic%		Sn:S
		Sn	S	
150	0.02	53.4	46.6	1.14
150	0.04	53.3	46.7	1.14
150	0.06	52.9	47.1	1.12
300	0.04	53.2	46.8	1.14

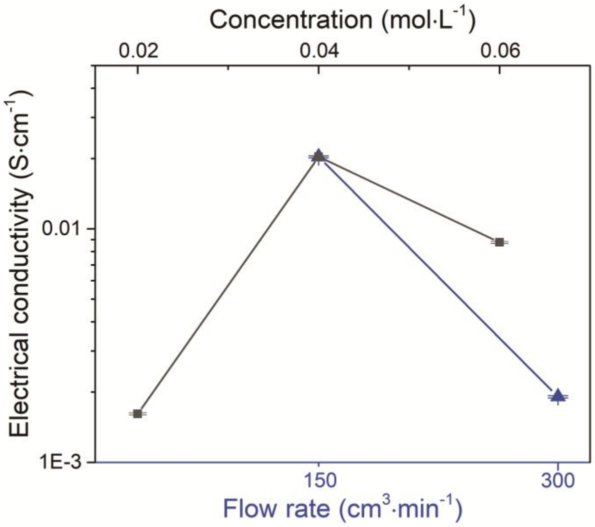

Figure S10. Room temperature electrical conductivity for SnS films deposited at a set temperature of 450 ℃ using different solution concentrations and flow rates. Uncertainty bars represent 1% and are comparable in size with the data points.

Table S7. EDX quantification results and Sn:S ratios for SnS films deposited using a flow rate of 150 $cm^3 \cdot min^{-1}$ and a solution concentration of 0.04 $mol \cdot L^{-1}$ at temperatures of 375 °C, 397 °C, 418 °C and 445 °C.

Deposition temperature (°C)	Atomic%		Sn:S
	Sn	S	
375	52.9	47.2	1.12

397	53.2	46.9	1.13
418	52.9	47.1	1.12
445	52. 7	47.3	1.11

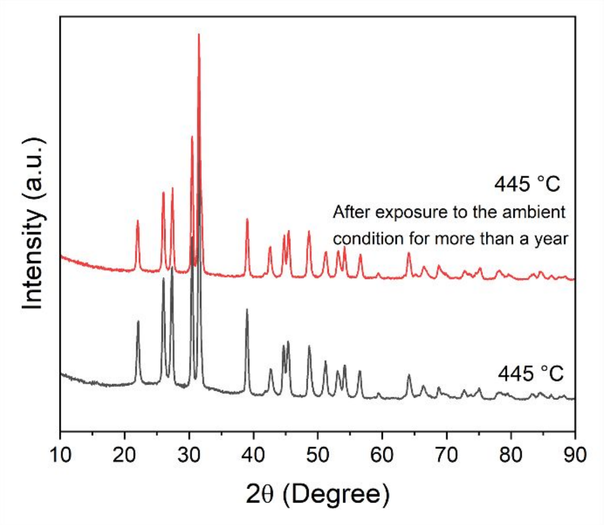

Figure S11. XRD patterns obtained immediately after deposition and after exposure to the ambient condition more than a year for the SnS film deposited at 445 ℃.

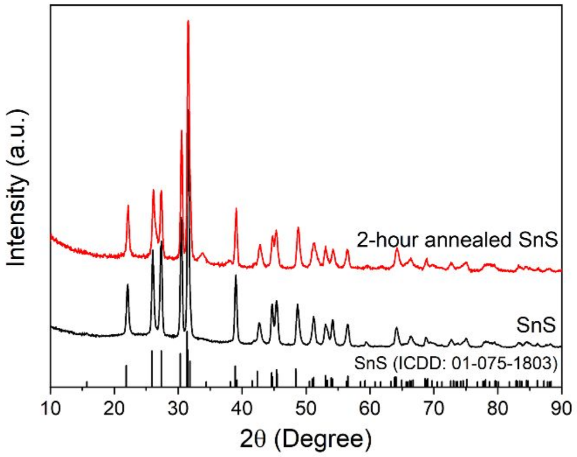

Figure S12. XRD patterns for the unannealed SnS films prepared at 445 °C and the SnS film prepared at 445 °C and annealed at 445 °C for 2 hours.

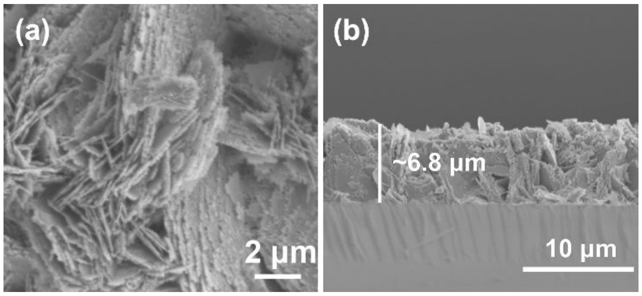

Figure S13. (a) SEM micrograph and (b) cross-sectional image for the annealed SnS film.

Table S8. The electrical conductivities for the unannealed and annealed SnS films.

Samples	Thickness (μm)	Electrical conductivity (S·cm^{-1})

SnS film prepared at 445 °C	6.5	0.19
SnS film prepared at 445 °C annealed at 445 °C for 2 hours	6.8	0.57

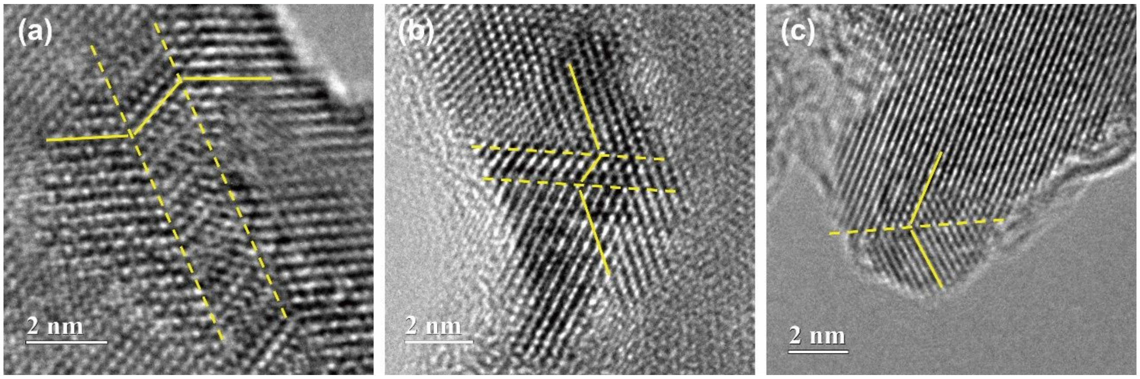

Figure S14. (a) – (c) HRTEM images of twin boundaries (yellow dash lines) for SnS film deposited at 445 ℃.

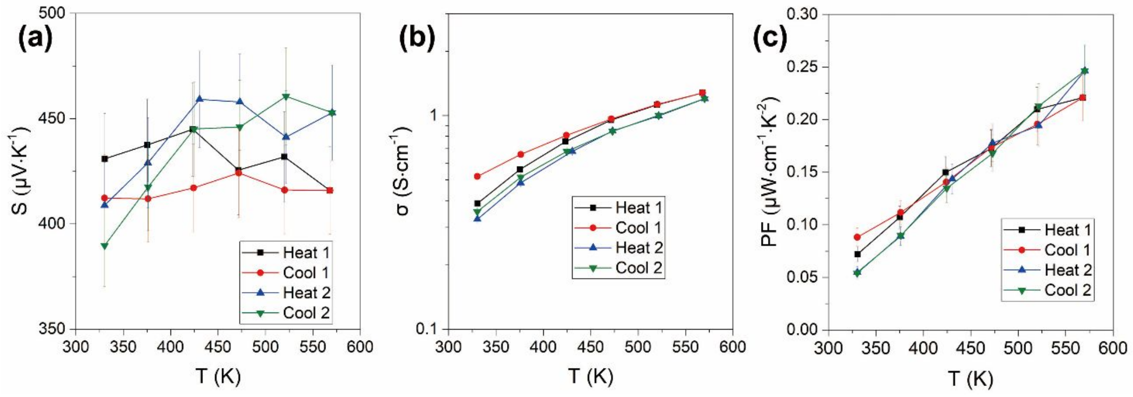

Figure S15. The temperature dependence of (a) Seebeck coefficients (*S*), (b) electrical conductivity (σ), and (c) power factor (*PF*) for SnS films deposited at 445 °C in heating and cooling cycles.

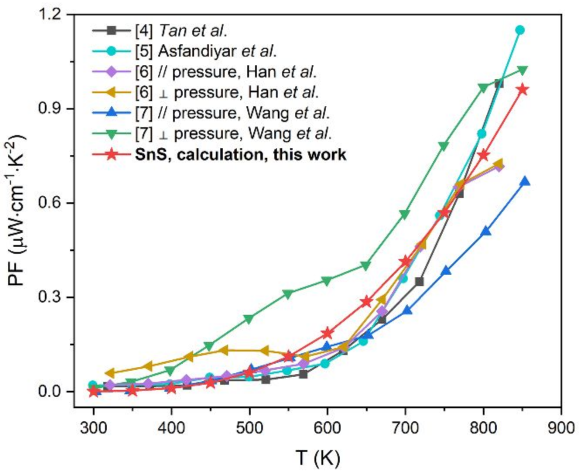

Figure S16. Comparison of the power factor (*PF*) for SnS as a function of temperature calculated in this work with experimental data reported for bulk SnS.[4-7]

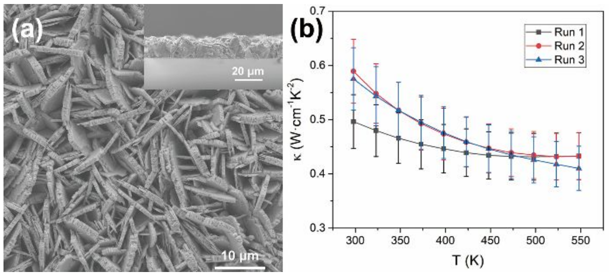

Figure S17. (a) SEM micrographs and cross sectional image for the SnS film deposited on the chip with Si_3N_4 membrane; the thickness of the SnS film is ~12.3 µm. (b) The temperature dependence of thermal conductivity for SnS film deposited at 445 °C; the measurement was repeated 3 times.

References:

(1) Linseis, V.; Völklein, F.; Reith, H.; Woias, P.; Nielsch, K. Platform for in-plane ZT measurement and Hall coefficient determination of thin films in a temperature range from 120 K up to 450 K. *J. Mater. Res.* **2016**, *31*, 3196-3204.
(2) Linseis, V.; Volklein, F.; Reith, H.; Nielsch, K.; Woias, P. Advanced platform for the in-plane ZT measurement of thin films. *Rev. Sci. Instrum.* **2018**, *89*, 015110.
(3) Lotgering, F. K. Topotactical reaction with ferrimagnetic oxides having hexagonal crystal structures. *J. Inorg. Nucl. Chem.* **1959**, *9*, 113-123.
(4) Tan, Q.; Zhao, L. D.; Li, J. F.; Wu, C. F.; Wei, T. R.; Xing, Z. B.; Kanatzidis, M. G. Thermoelectrics with earth abundant elements: low thermal conductivity and high thermopower in doped SnS. *J. Mater. Chem. A* **2014**, *2*, 17302-17306.
(5) Asfandiyar; Cai, B.; Zhao, L. D.; Li, J. F. High thermoelectric figure of merit ZT > 1 in SnS polycrystals. *J. Materiomics* **2020**, *6*, 77-85.
(6) Han, Y. M.; Zhao, J.; Zhou, M.; Jiang, X. X.; Leng, H. Q.; Li, L. F. SnSeS Thermoelectric performance of SnS and SnS–SnSe solid solution. *J. Mater. Chem. A* **2015**, *3*, 4555-4559.
(7) Wang, C.; Chen, Y.; Jiang, J.; Zhang, R.; Niu, Y.; Zhou, T.; Xia, J.; Tian, H.; Hu, J.; Yang, P. Improved thermoelectric properties of SnS synthesized by chemical precipitation. *RSC Adv.* **2017**, *7*, 16795-16800.
